# Computer-Aided Analysis of Multiple SARS-CoV-2 Therapeutic Targets: Identification of Potent Molecules from African Medicinal Plants

**DOI:** 10.1155/2020/1878410

**Published:** 2020-09-12

**Authors:** Franklyn Nonso Iheagwam, Solomon Oladapo Rotimi

**Affiliations:** ^1^Department of Biochemistry, College of Science and Technology, Covenant University, Canaanland, P.M.B. 1023, Ota, Ogun, Nigeria; ^2^Covenant University Public Health and Wellness Research Cluster (CUPHWERC), College of Science and Technology, Covenant University, Canaanland, P.M.B. 1023, Ota, Ogun, Nigeria

## Abstract

The COVID-19 pandemic, which started in Wuhan, China, has spread rapidly over the world with no known antiviral therapy or vaccine. Interestingly, traditional Chinese medicine helped in flattening the pandemic curve in China. In this study, molecules from African medicinal plants were analysed as potential candidates against multiple SARS-CoV-2 therapeutic targets. Sixty-five molecules from the ZINC database subset (AfroDb Natural Products) were virtually screened with some reported repurposed therapeutics against six SARS-CoV-2 and two human targets. Molecular docking, druglikeness, absorption, distribution, metabolism, excretion, and toxicity (ADMET) of the best hits were further simulated. Of the 65 compounds, only three, namely, 3-galloylcatechin, proanthocyanidin B1, and luteolin 7-galactoside found in almond (*Terminalia catappa*), grape (*Vitis vinifera*), and common verbena (*Verbena officinalis*), were able to bind to all eight targets better than the reported repurposed drugs. The findings suggest these molecules may play a role as therapeutic leads in tackling this pandemic due to their multitarget activity.

## 1. Introduction

Coronaviruses (CoVs) are members of Coronaviridae family belonging to the order Nidovirales. They are enveloped viruses of about 60 to 140 nm in diameter with positive-sense single-stranded RNA genome (+ssRNA) classified into four genera, namely, alpha (*α*), beta (*β*), gamma (*γ*), and delta (*δ*) [1]. These viruses have spikes protruding from their surface, giving them a crown-like structure (hence, the name corona), which makes them bind to the human lower respiratory system [2]. Since the turn of the 21^st^ century, CoVs have caused several pandemics that are not only of significant public health concerns but also distort socioeconomic activities in infected regions [3]. In 2003, Severe Acute Respiratory Syndrome (SARS) was identified in Guangdong, China, with SARS-CoV as the causative pathogen, while the Middle East respiratory syndrome (MERS) caused by MERS-CoV resurfaced a decade later in Jeddah, Saudi Arabia [4]. These zoonotic pathogens belong to the *β*-CoV genus, with pneumonia and acute respiratory distress syndrome (ARDS) as prominent symptoms [5]. In Wuhan, China, a novel pandemic initially known as 2019 novel coronavirus (2019-nCoV) was reported in December 2019. It was later called coronavirus disease (COVID-19) by the World Health Organization (WHO) on 11 February 2020 [6]. The disease exhibits similar symptoms with SARS, consequently making the International Committee on Taxonomy of Viruses (ICTV) name the viral pathogen severe acute respiratory syndrome coronavirus 2 (SARS-CoV-2) [7]. Irrespective of the numerous ways and policies to contain this pandemic, it has continued to spread worldwide with an increase in its related mortality. The WHO situation report as of 8 June 2020 shows 6,931,000 confirmed cases with 400,857 reported deaths worldwide. In Africa, 135,412 confirmed cases with 3,236 deaths were reported with the transmission classified majorly as community-based [8]. The novelty of this disease means there is no antiviral therapy or vaccine to combat it. Nonetheless, the severity of this disease has led to its prioritisation and increased research on the disease and the virus by researchers worldwide, leading to a better understanding of its aetiology, pathogenesis, management, and treatment [1]. Recently, lopinavir, ritonavir, remdesivir, chloroquine, hydroxychloroquine, camostat, and nafamostat, to mention a few, have been proposed as potential drug candidates that could be repurposed to combat this pandemic [9–12]. Traditional Chinese Medicine (TCM) and Ayurveda system of medicine have been attributed to play a part in the flattening of the pandemic curve in China with about 60,000 people treated with TCM [13, 14]. Theoretically, the secondary active metabolites in these natural products may have been responsible for the attributed success of TCM against COVID-19 in China.

Computational method approaches are important techniques which efficiently filter, screen, select, and identify potential leads for drug development from diverse chemical databases [15]. Numerous computational screening analyses have been carried out to screen and identify drug candidates with therapeutic and prophylactic potential against various proteins of SARS-CoV-2 from the ZINC database [16–20]. Network analysis leaning algorithm (machine learning, deep learning, and artificial intelligence (AI)) approach based on a fully connected neural network in combination with virtual screening methods as well as network-based meta-analysis has been utilised in investigating potential anti-SARS-CoV-2 leads from ZINC database [21–25]. The Unites States Food and Drug Administration- (USFDA-) approved drugs [26], drugbank [27, 28], traditional Ayurvedic, Chinese and natural medicine [20, 28–31], dark chemical matter, and fooDB [25] are some of the ZINC database subsets that have been rigourously screened for molecules to combat SARS-CoV-2 with main protease, RNA-dependent RNA polymerase, and angiotensin-converting enzyme-2 as the major therapeutic targets. Despite the volume of research on computational screening analyses from different databases, there is a paucity of information on small molecules from African medicinal plants and other therapeutic targets that can help combat SARS-CoV-2. Hence, this study analysed a plethora of natural products (NPs) from African medicinal plants with known bioactivities in human as therapeutic candidates targeting and inhibiting SARS-CoV-2 RNA synthesis, replication, structural protein function, and host-specific receptors/enzymes.

## 2. Materials and Methods

### 2.1. Ligand Modelling

A total of 65 compounds from African medicinal plants with known bioactivities in human were downloaded as a subset of AfroDb Natural Products (http://zinc15.docking.org/catalogs/afronp/substances/subsets/in-man/) as shown in Table S1. At the same time, lopinavir, ritonavir, remdesivir, chloroquine, hydroxychloroquine, camostat, and nafamostat which are proposed drug candidates in treating COVID-19 were downloaded from PubChem (http://www.pubchem.ncbi.nlm.nih.gov) in .sdf format and used as standards. Conversion to their three-dimensional (3D) structures, force field generation, and the addition of nonpolar hydrogen and Gasteiger charges were carried out using Open Babel v2.3.2 [32], LigParGen [33], and AutoDock4.2 [34, 35], respectively.

### 2.2. Protein Retrieval, Preparation, and Binding Pocket Prediction

The protein structures of SARS-CoV-2 papain-like protease (PLpro), main/3-chymotrypsin-like protease (3CLpro), RNA-dependent RNA polymerase (RdRp), helicase, 2-O-methyltransferase (2OMT), spike receptor-binding domain (S-RBD), human angiotensin-converting enzyme 2 (ACE2), and human type-II transmembrane serine protease (TMPRSS2) with PDB codes 6w9c, 6lu7, 6m71, 6w4h, 6m17.E, 6m17.B, and 5ce1, respectively, were retrieved from protein data bank (http://www.rcsb.org). Experimental SARS-CoV-2 helicase structure is yet to be deposited in the protein data bank; hence, a homology structure was modelled as described by Iheagwam and Ogunlana [36]. Briefly, SARS-CoV-2 helicase sequence was acquired from UniProt (http://www.uniprot.org) via the UniProt identifier (P0DTD1) with a feature identifier (PRO_0000449630). The FASTA sequence was inputted in SWISS-MODEL [37] to generate a theoretical model which was evaluated using PROCHECK, ERRAT [38], and Verify 3D [39]. 3Drefine was used to minimise the energy levels of all protein crystal structures [40]. All therapeutic targets were run on the DogSiteScorer server for prediction of their molecular druggable pocket [41].

### 2.3. Structure Based-Virtual Screening and Molecular Docking

iGEMDOCK v2.1 was used to screen the ligands in the binding pockets of the proteins with population size 300, 80 generations, and 10 solutions as the set screening parameters [42]. Top ranking ligands present across all eight proteins [8] were further subjected to molecular docking using AutoDock Vina [43]. Gasteiger charges were computed, polar hydrogen was assigned, and grid map was set at 1 Å space for PLpro, 3CLpro, RdRp, helicase, 2OMT, S-RBD, ACE2, and TMPRSS2 protein crystal structures using Autodock 4.2 as shown in Table 1 before molecular docking was carried out [34, 35].

### 2.4. Physicochemical, Pharmacokinetic, and Toxicity Evaluation

Physicochemical, pharmacokinetic, and toxicity parameters of the identified hit therapeutic molecules were predicted using SwissADME [44] and ADMETlab [45].

## 3. Results and Discussion

### 3.1. Homology Modelling

The SARS-CoV-2 helicase FASTA sequence (601 amino acid residues) which was inputted into SWISS-MODEL generated five helicase homology models from 5 templates, namely, 6jyt, 5wwp, 4non, 5ftf, and 6sje. However, the model built using 5wwp as template was selected based on its global model quality estimate, QMEAN, template resolution, sequence identity, similarity, and coverage over other templates (Table S2, Figure S1). In SWISS-MODEL, structural information of templates is extracted based on OpenStructure comparative modelling engine to provide complete stoichiometry and generate a 3D homology model structure [37]. According to Xiang [46], if the sequence identity similarity of 30% upward is shared between target and template, the homology model is considered dependable and successful. With a 72.2% similarity and QMEAN of −1.72, the selected model was not only successful but might have structural similarity and behaviour with experimental models [47]. Upon energy minimisation of the modelled helicase, a root-mean-square deviation (RMSD) score of 0.387 and 0.588 Å was observed when superimposed with the modelled helicase and 5wwp, respectively (Figure S2). This observation suggests the homology model has a high similarity and structural orderliness with the template as corroborated by studies on TMPRSS2 and dipeptidyl peptidase 4 [17, 36]. The model also had good stereochemical quality as conferred by the Ramachandran plot with reduced noise as shown in Table S3 with a 3D verification score of 94.7% and quality factor of 88.14% further corroborating the reliability of the model (Figures S3 and S4, respectively).

### 3.2. Structure-Based Virtual Screening and Molecular Docking

In the course of drug discovery, structure-based virtual screening is a computational approach utilised to identify promising novel small chemical ligands from curated chemical compound databases with potential activity against drug targets [48].  Table S4 shows the virtual screening results of molecules downloaded from ZINC database subset (AfroDb Natural Products) against multiple SARS-CoV-2 targets using iGEMDOCK v2.1. The total binding energy ranged from −51.3229 to −127.014, 156.29 to −122.363, −38.3427 to −107.434, 71.1802 to −105.299, −29.8219 to −114.424, −36.3285 to −81.2413, −35.5357 to −91.4829, and 268.746 to −133.244 kcal/mol for PLpro, 3CLpro, RdRp, helicase, 2OMT, S-RBD, human ACE2, and human TMPRSS2, respectively. Looking at the top 15 scoring ligands, only ZINC 3978503, ZINC 5085289 and ZINC 40422816 also known as 3-galloylcatechin, proanthocyanidin B1, and luteolin 7-galactoside, respectively, appeared across all therapeutic targets as the 3^rd^, 10^th^, and 5^th^ respective ranking molecule for PLpro; 1^st^, 3^rd^, and 9^th^ respective ranking molecule for 3CLpro; 2^nd^, 4^th^, and 9^th^ respective ranking molecule for RdRp; 4^th^, 1^st^, and 3^rd^ respective ranking molecule for helicase; 3^rd^, 4^th^, and 1^st^ respective ranking molecule for 2OMT; 8^th^, 2^nd^, and 12^th^ respective ranking molecule for S-RBD; 10^th^, 1^st^, and 7^th^ respective ranking molecule for ACE2; and 4^th^, 5^th^, and 10^th^ respective ranking molecule for TMPRSS2 (Table S4). These three compounds were further docked using Autodock Vina in the binding pocket of the therapeutic targets predicted by DogSiteScorer, as shown in Figure S5. Pocket and subpocket detection and analysis tools are useful for assessing the druggability of therapeutic targets. DoGSiteScorer detects druggable pockets using a support vector machine based on the integration of grid-based method and Gaussian filter difference [41]. Pocket detection is usually done prior to molecular docking simulations. The selected hit ligands exhibited better Autodock binding fitness than the proposed COVID-19 treatment drug candidates in the binding pocket of PLpro (−5.7, −4.1, and −4.6 kcal/mol, respectively), 3CLpro (−6.3, −5.1, and −5.6 kcal/mol, respectively), RdRp (−8.7, −9.8, and −8.5 kcal/mol, respectively), helicase (−9.8, −10.3, and −9.2 kcal/mol, respectively), 2OMT (−8.6, −10.5, and −9.6 kcal/mol, respectively), S-RBD (−5.2, −6.4, and −6.1 kcal/mol, respectively), ACE2 (−8.5, −9.7, and −8.9 kcal/mol, respectively), and TMPRSS2 (−8.4, −8.9, and −8.9 kcal/mol, respectively) (Table 2). Interestingly, none of them were able to target the S-RBD-ACE2 interface (result not shown). In AutoDock Vina, ligands with more electronegative binding energy are ranked higher and believed to have better binding fitness [43]. 3-Galloylcatechin, proanthocyanidin B1, and luteolin 7-galactoside all had lower AutoDock Vina scores than the proposed standards in most of the targets, suggesting better binding fitness, lower inhibition constant, and better experimental activity values [49]. 3-Galloylcatechin is an extremely weak base flavonoid with almond (*Terminalia catappa*) and grapes (*Vitis vinifera*) as rich sources [50]. Proanthocyanidin B1-like 3-galloylcatechin is also a flavonoid made up of (−)-epicatechin and (+)-catechin units, joined by a *β*-interflavanyl bond. It is found in cocoa powder and grapes at high concentration [51]. Luteolin 7-galactoside is a flavonoid glycoside found in fruits, herbs, and spices such as common verbena (*Verbena officinalis*) [52]. The antiviral activity of 3-galloylcatechin and proanthocyanidin B1 has previously been reported [53, 54]. Ghosh and Chakraborty [55] and Nguyen and Woo [56] in their respective studies have identified gallocatechin gallate, a closely related molecule to 3-galloylcatechin, as a candidate molecule against SARS-CoV 3CLpro. Competitive inhibition of this enzyme was the proposed mode of gallocatechin gallate action. The inhibitory activity of procyanidin B1 on SARS-CoV infection has been reported by Zhuang et al. [57]. Though procyanidin B1 mode of action was not elucidated, transferrin receptor and ACE2 expression were found not to play a role in the inhibitory property. Luteolin 7-galactoside, on the other hand, does not have any reported antiviral activity, but rather its effectiveness in treating sore throats, respiratory tract diseases, and its anti-inflammatory activity is well established [58, 59]. The carbohydrate moiety stereoisomer, luteolin 7-glucoside, on the other hand, has been reported to possess antiviral activity [60, 61]. Luteolin was recently reported to possess possible strong antiviral activity against SARS-CoV-2 as a result of its low binding energy in the active site of some therapeutic targets [62].

**Figure 1:**
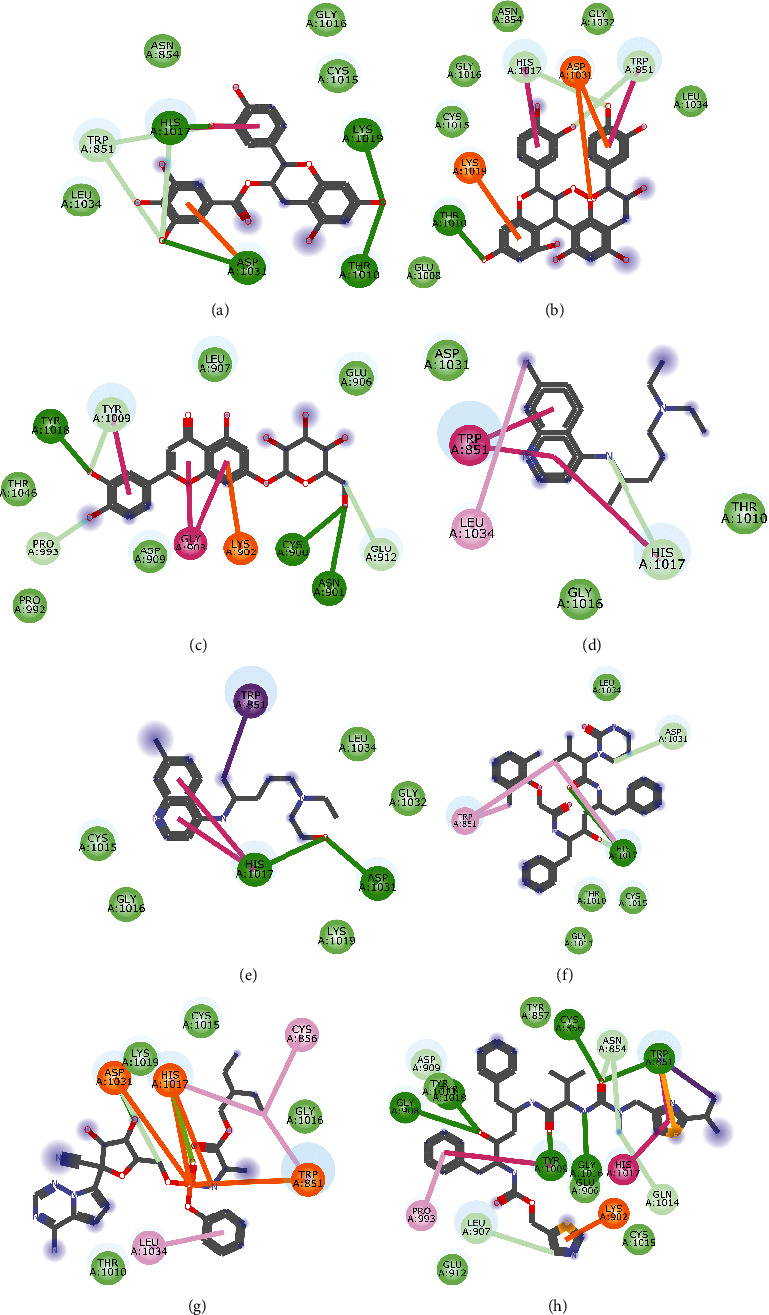
2D representation of (a) 3-galloylcatechin, (b) proanthocyanidin B1, (c) luteolin 7-galactoside, (d) chloroquine, (e) hydroxychloroquine, (f) lopinavir, (g) remdesivir, and (h) ritonavir in the binding pocket of PLpro. Hydrogen, carbon-hydrogen, and *π* bonds are depicted as green, light blue, and any other coloured (purple, magenta, orange, turquoise blue, pink, and yellow) lines, while Van der Waal interactions appear as light green circles.

**Figure 2:**
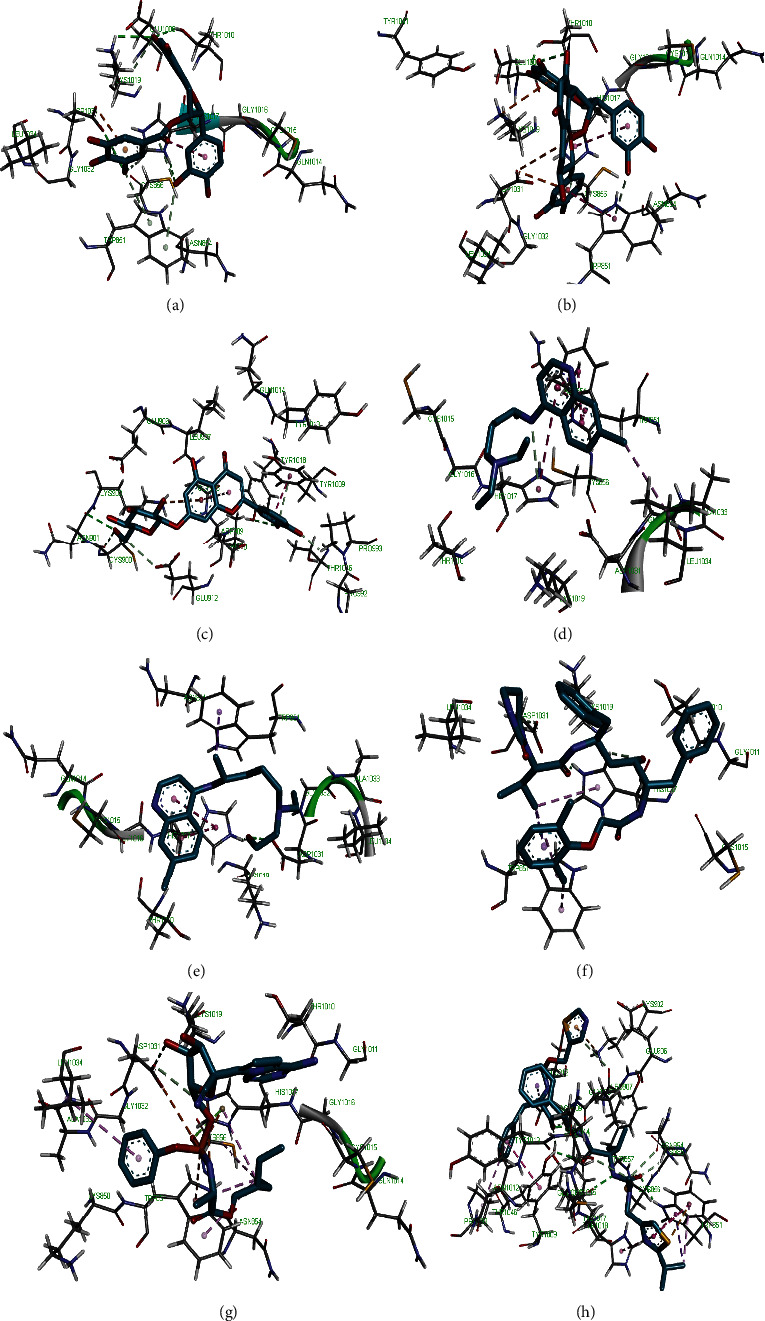
3D representation of (a) 3-galloylcatechin, (b) proanthocyanidin B1, (c) luteolin 7-galactoside, (d) chloroquine, (e) hydroxychloroquine, (f) lopinavir, (g) remdesivir, and (h) ritonavir in the binding pocket of PLpro.

**Figure 3:**
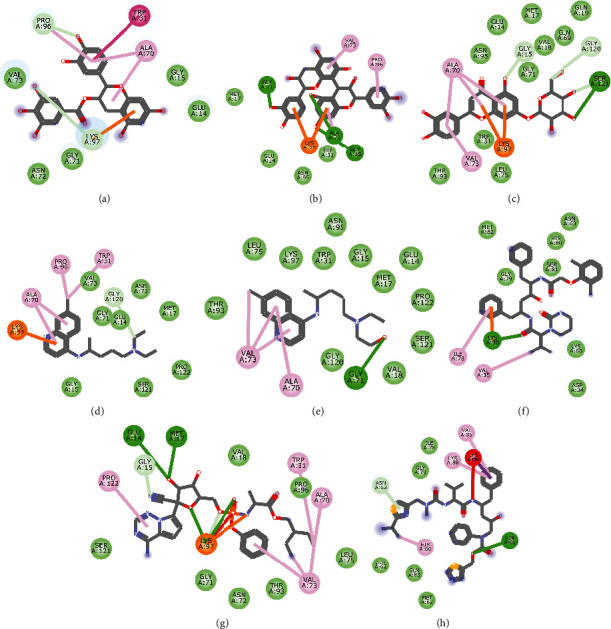
2D representation of (a) 3-galloylcatechin, (b) proanthocyanidin B1, (c) luteolin 7-galactoside, (d) chloroquine, (e) hydroxychloroquine, (f) lopinavir, (g) remdesivir, and (h) ritonavir in the binding pocket of 3CLpro. Hydrogen, carbon-hydrogen, and unfavourable and *π* bonds are depicted as green, light blue, red, and any other coloured (purple, magenta, orange, and pink) lines, while Van der Waal interactions appear as light green circles.

**Figure 4:**
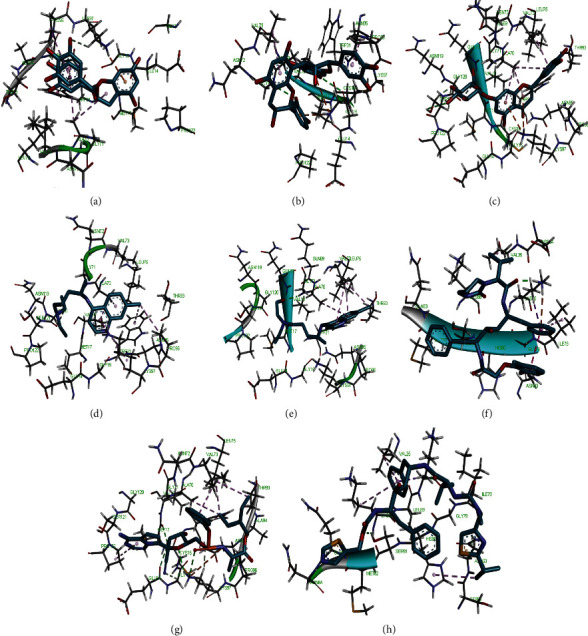
3D representation of (a) 3-galloylcatechin, (b) proanthocyanidin B1, (c) luteolin 7-galactoside, (d) chloroquine, (e) hydroxychloroquine, (f) lopinavir, (g) remdesivir, and (h) ritonavir in the binding pocket of 3CLpro.

**Figure 5:**
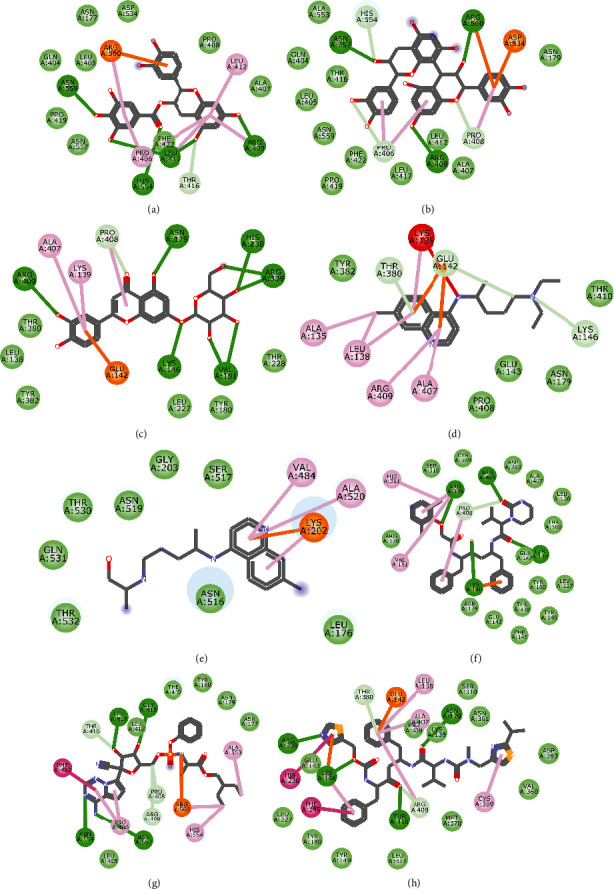
2D representation of (a) 3-galloylcatechin, (b) proanthocyanidin B1, (c) luteolin 7-galactoside, (d) chloroquine, (e) hydroxychloroquine, (f) lopinavir, (g) remdesivir, and (h) ritonavir in the binding pocket of helicase. Hydrogen, carbon-hydrogen, and unfavourable and *π* bonds are depicted as green, light blue, red, and any other coloured (purple, magenta, orange, turquoise blue, pink, and yellow) lines, while Van der Waal interactions appear as light green circles.

**Figure 6:**
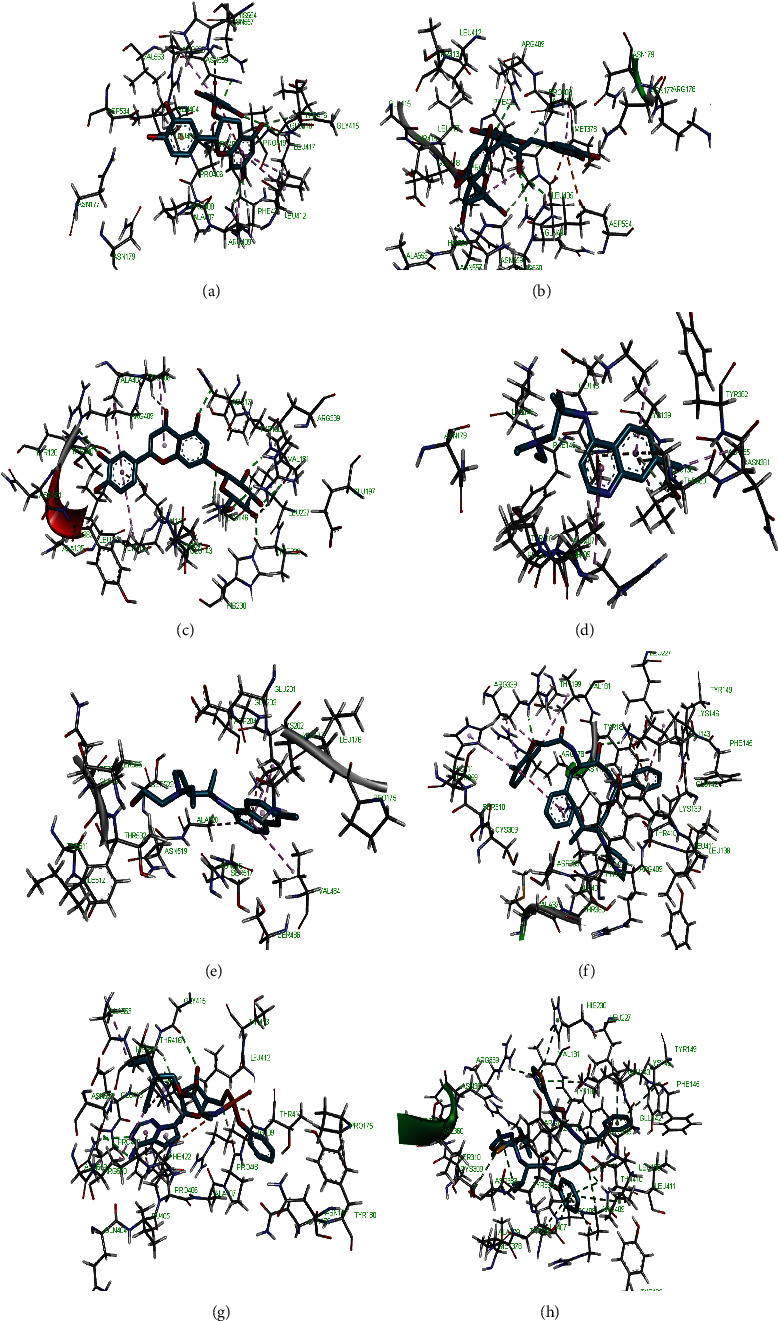
3D representation of (a) 3-galloylcatechin, (b) proanthocyanidin B1, (c) luteolin 7-galactoside, (d) chloroquine, (e) hydroxychloroquine, (f) lopinavir, (g) remdesivir, and (h) ritonavir in the binding pocket of helicase.

**Figure 7:**
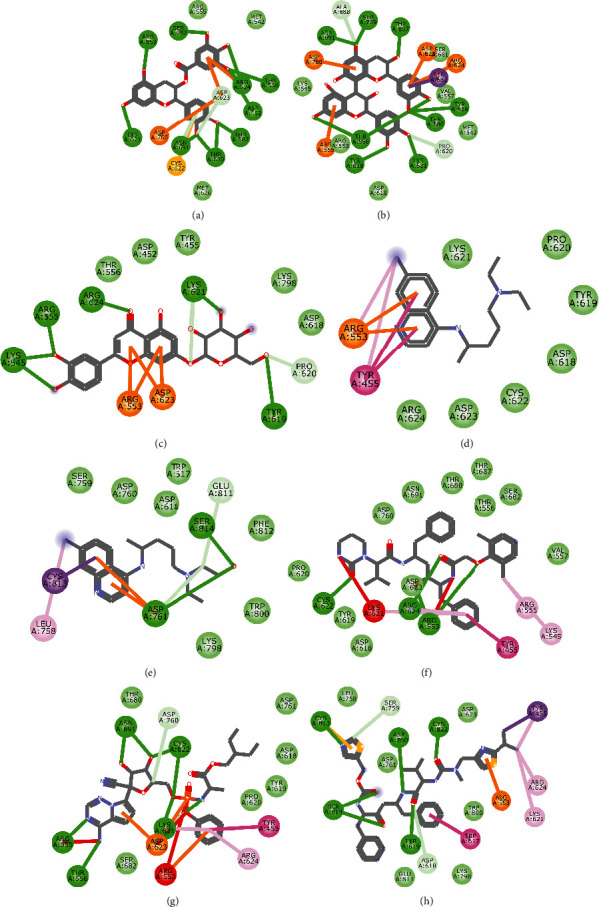
2D representation of (a) 3-galloylcatechin, (b) proanthocyanidin B1, (c) luteolin 7-galactoside, (d) chloroquine, (e) hydroxychloroquine, (f) lopinavir, (g) remdesivir, and (h) ritonavir in the binding pocket of RdRp. Hydrogen, carbon-hydrogen, unfavourable, and *π* bonds are depicted as green, light blue, red, and any other coloured (purple, magenta, orange, turquoise blue, pink, and yellow) lines, while Van der Waal interactions appear as light green circles.

**Figure 8:**
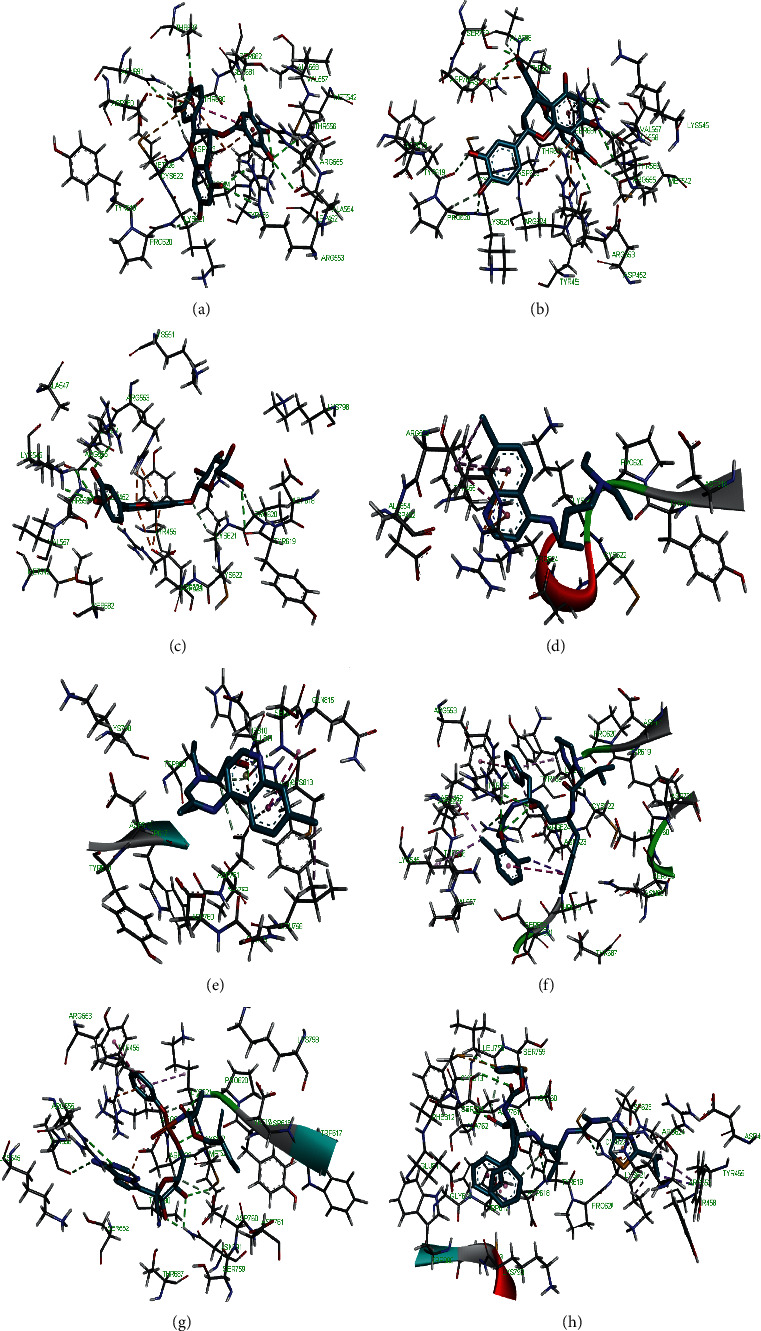
3D representation of (a) 3-galloylcatechin, (b) proanthocyanidin B1, (c) luteolin 7-galactoside, (d) chloroquine, (e) hydroxychloroquine, (f) lopinavir, (g) remdesivir, and (h) ritonavir in the binding pocket of RdRp.

**Figure 9:**
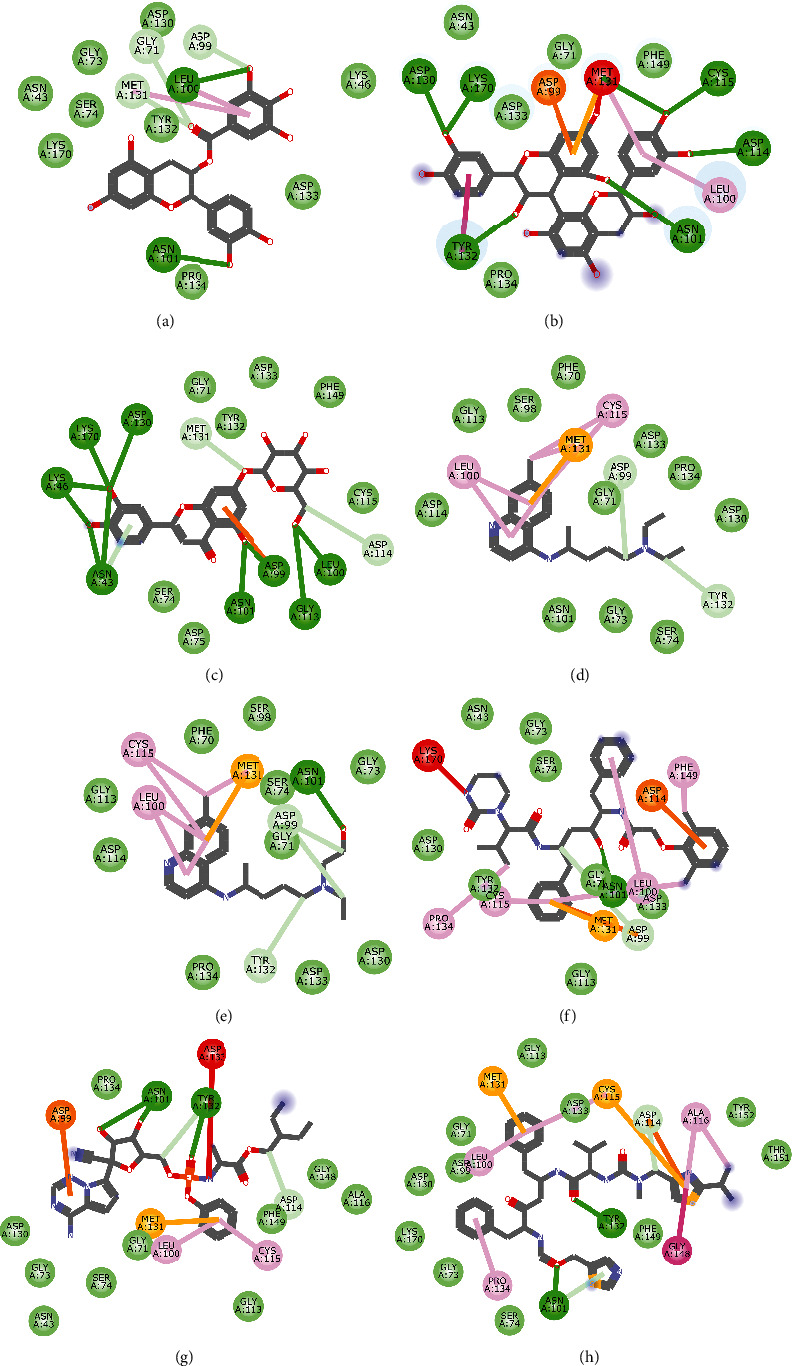
2D representation of (a) 3-galloylcatechin, (b) proanthocyanidin B1, (c) luteolin 7-galactoside, (d) chloroquine, (e) hydroxychloroquine, (f) lopinavir, (g) remdesivir, and (h) ritonavir in the binding pocket of 2OMT. Hydrogen, carbon-hydrogen, unfavourable, and *π* bonds are depicted as green, light blue, red, and any other coloured (purple, magenta, orange, turquoise blue, pink, and yellow) lines, while Van der Waal interactions appear as light green circles.

**Figure 10:**
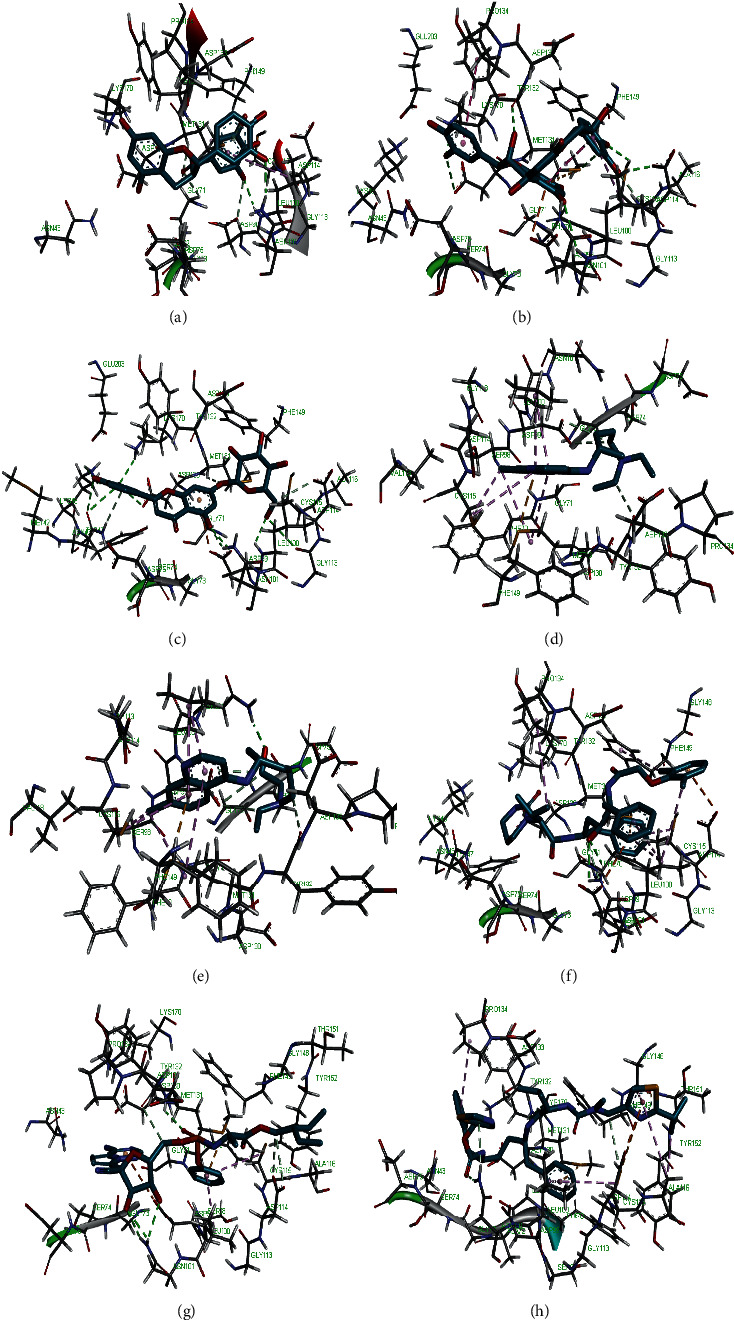
3D representation of (a) 3-galloylcatechin, (b) proanthocyanidin B1, (c) luteolin 7-galactoside, (d) chloroquine, (e) hydroxychloroquine, (f) lopinavir, (g) remdesivir, and (h) ritonavir in the binding pocket of 2OMT.

**Figure 11:**
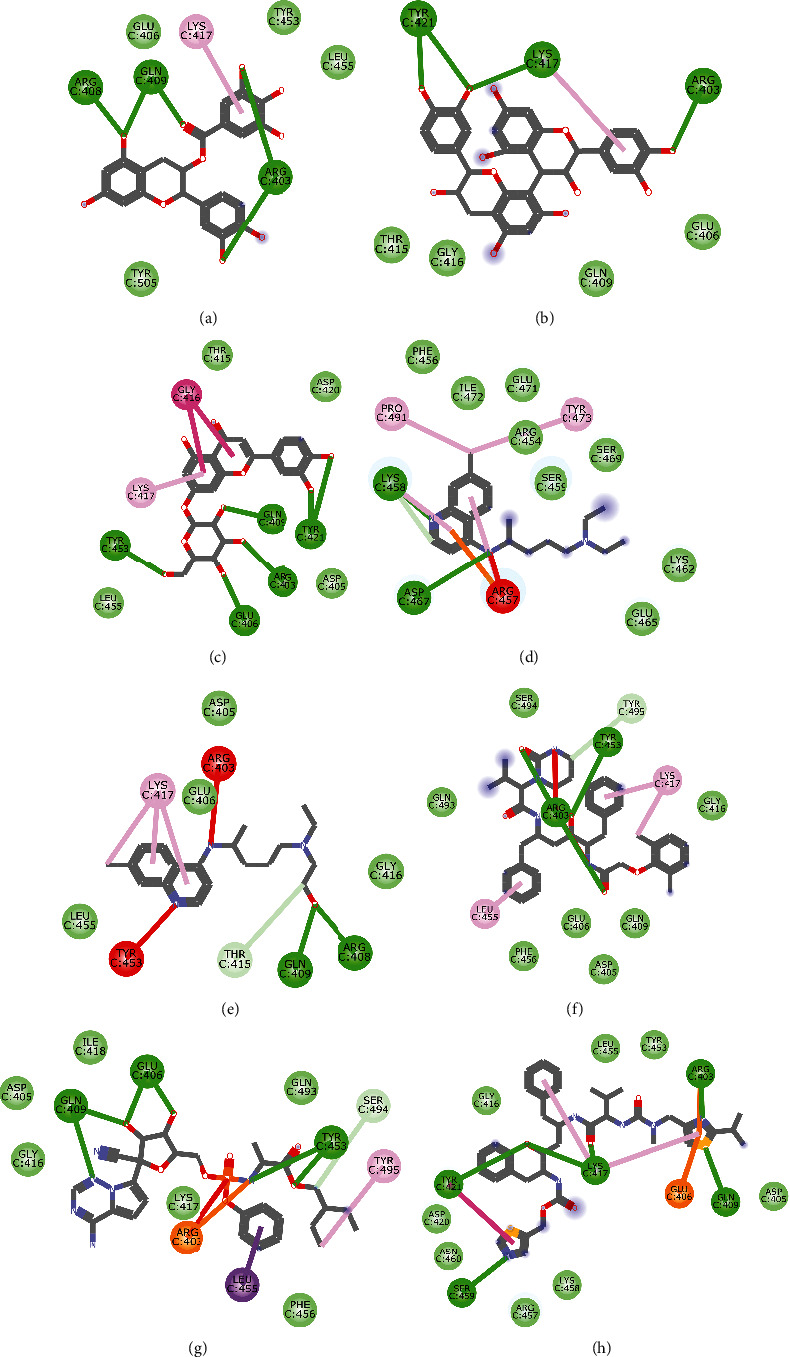
2D representation of (a) 3-galloylcatechin, (b) proanthocyanidin B1, (c) luteolin 7-galactoside, (d) chloroquine, (e) hydroxychloroquine, (f) lopinavir, (g) remdesivir, and (h) ritonavir in the binding pocket of S-RBD. Hydrogen, unfavourable, and *π* bonds are depicted as green, red, and any other coloured (purple, magenta, orange, and pink) lines, while Van der Waal interactions appear as light green circles.

**Figure 12:**
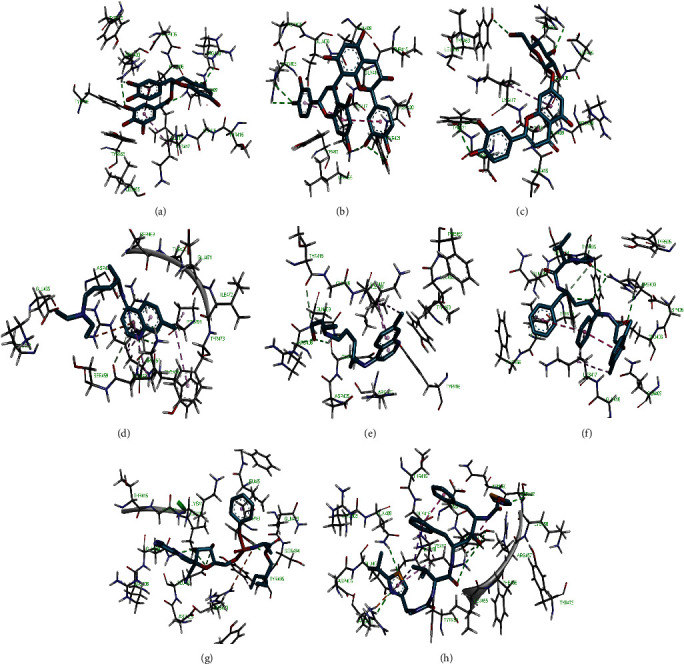
3D representation of (a) 3-galloylcatechin, (b) proanthocyanidin B1, (c) luteolin 7-galactoside, (d) chloroquine, (e) hydroxychloroquine, (f) lopinavir, (g) remdesivir, and (h) ritonavir in the binding pocket of S-RBD.

**Figure 13:**
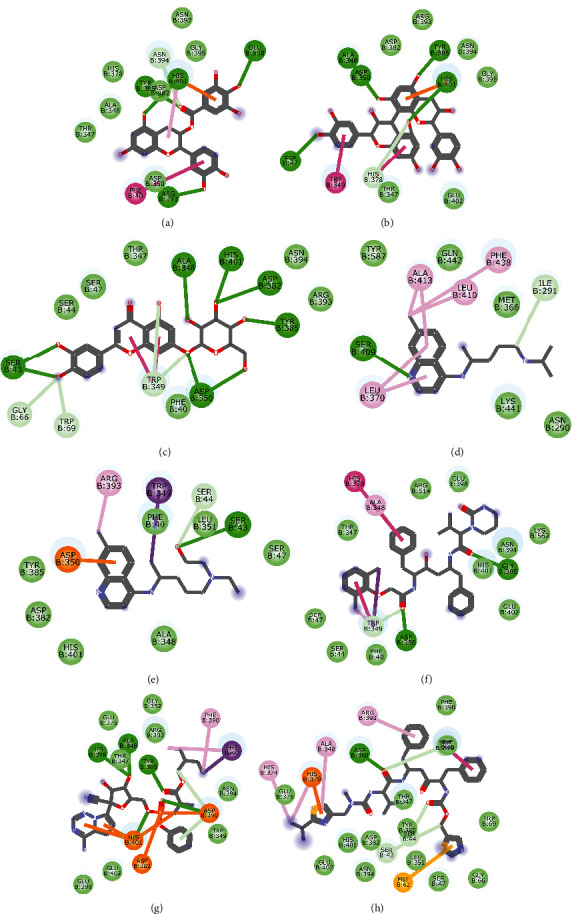
2D representation of (a) 3-galloylcatechin, (b) proanthocyanidin B1, (c) luteolin 7-galactoside, (d) chloroquine, (e) hydroxychloroquine, (f) lopinavir, (g) remdesivir, and (h) ritonavir in the binding pocket of ACE2. Hydrogen and *π* bonds are depicted as green and any other coloured (purple, magenta, orange, turquoise blue, pink, and yellow) lines, while Van der Waal interactions appear as light green circles.

**Figure 14:**
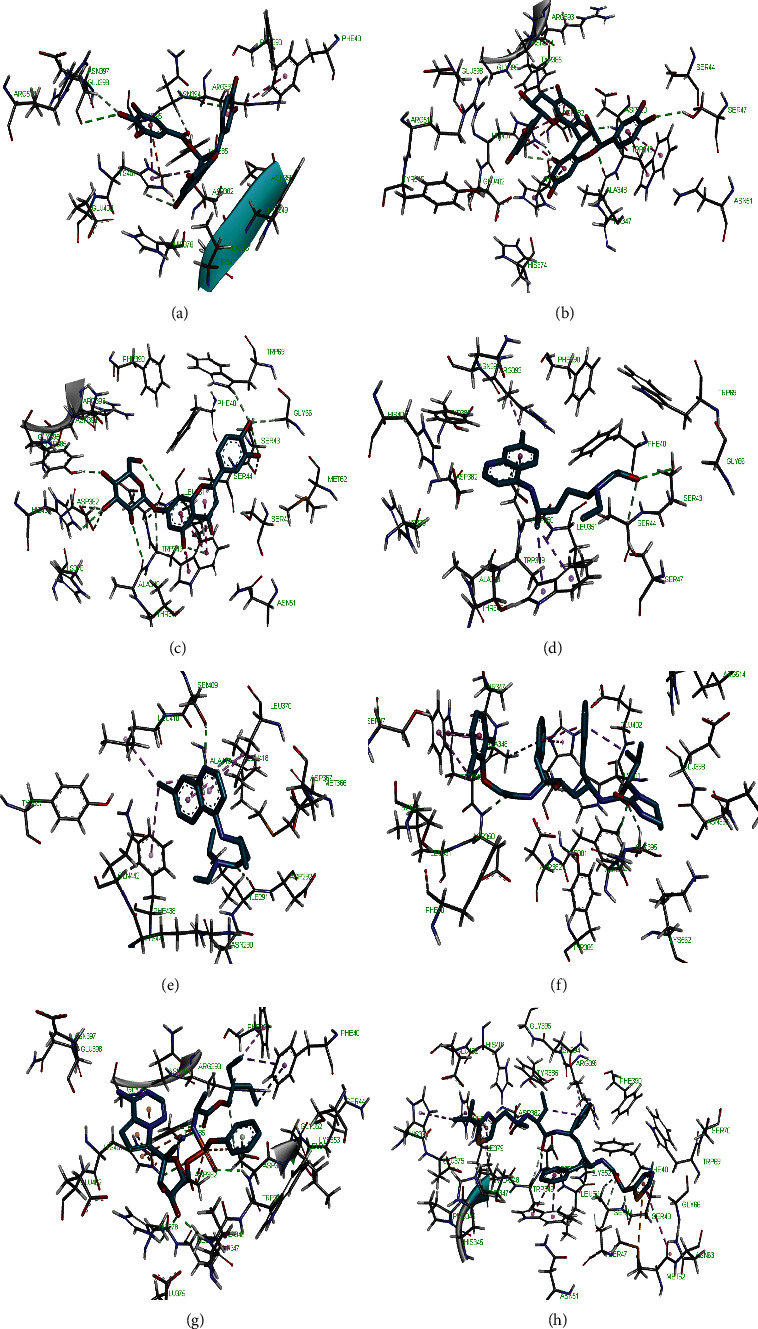
3D representation of (a) 3-galloylcatechin, (b) proanthocyanidin B1, (c) luteolin 7-galactoside, (d) chloroquine, (e) hydroxychloroquine, (f) lopinavir, (g) remdesivir, and (h) ritonavir in the binding pocket of ACE2.

**Figure 15:**
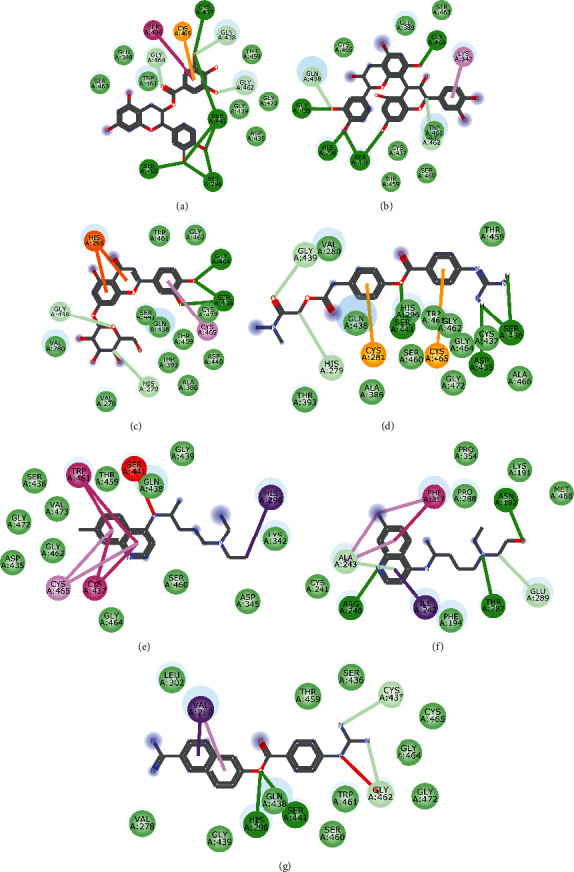
2D representation of (a) 3-galloylcatechin, (b) proanthocyanidin B1, (c) luteolin 7-galactoside, (d) camostat, (e) chloroquine, (f) hydroxychloroquine, and (g) nafamostat in the binding pocket of TMPRSS2. Hydrogen, carbon-hydrogen, and *π* bonds are depicted as green, light blue, and any other coloured (purple, magenta, orange, turquoise blue, pink, and yellow) broken lines, while Van der Waal interactions appear as light green circles.

**Figure 16:**
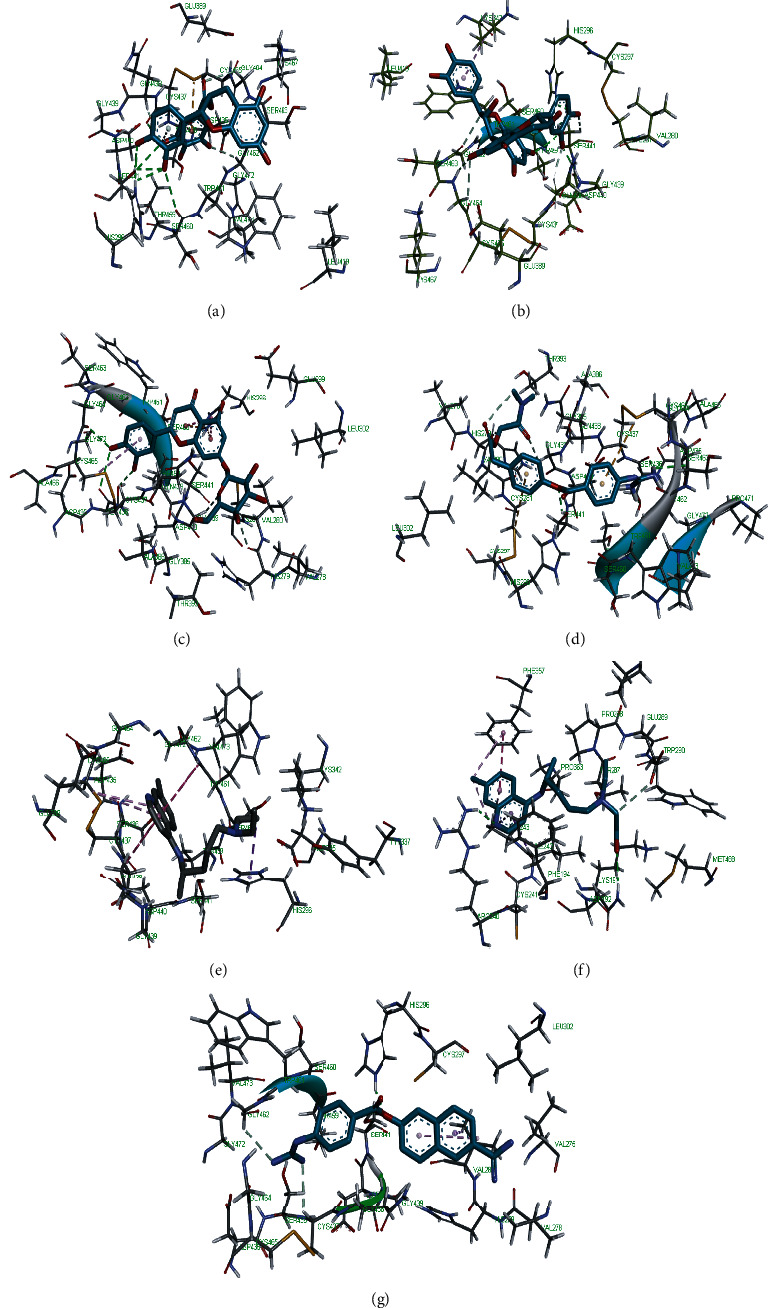
3D representation of (a) 3-galloylcatechin, (b) proanthocyanidin B1, (c) luteolin 7-galactoside, (d) camostat, (e) chloroquine, (f) hydroxychloroquine, and (g) nafamostat in the binding pocket of TMPRSS2.

Molecular docking simulation predicts the binding properties and interactions between a ligand and its target [63]. Looking at the binding mode and molecular interaction of the hit ligands in the binding pocket of SARS-CoV-2 therapeutic targets (PLpro, 3CLpro, RdRp, helicase, 2OMT, S-RBD, ACE2, and TMPRSS2) as simulated by Autodock Vina, conventional hydrogen bonds, carbon-hydrogen bond, Van der Waal forces, and various pi (*π*) interactions were responsible for stabilising the ligand interactions (Figures 1–16). HIS1017 and CYS1015 were noteworthy common amino acid residues that interacted via hydrogen bond and Van der Waal interaction, respectively, with all the hit ligands and proposed drugs in the binding site of PLpro (Figure 1). In the binding pocket of 2OMT as depicted in Figures 9-10, ASN101 (hydrogen bond), GLY71, ASP133 (Van der Waal forces), LEU100, and MET131 (*π* interactions) were the common amino acid residues that interacted with the hit molecules and proposed standards to stabilise them in the binding site while ASP618 (Van der Waal interaction) was synonymous in the binding site of RdRp (Figures 7-8). These amino acid residues could be targeted as possible therapeutic targets in the inhibition of these proteins due to their noncovalent stabilising activity in the druggable pocket [64]. HIS contains an imidazole side chain with acid-base properties which is essential in the catalytic mechanism of enzymes where it is found [65]. For 3CLpro, helicase, S-RBD, ACE2, and TMPRSS2, the amino acid residues responsible for stabilising the molecules in the binding site were not consistent for the ligands and proposed standards (Figures 3–6 and 11, 12–15, and 16, respectively).

All ligands and proposed standards were able to bind properly in the binding pocket of the therapeutic targets as predicted by DogSiteScorer after the docking simulation by Autodock Vina (Figure S5). On the other hand, luteolin 7-galactoside and ritonavir bound to the allosteric site of PLpro with ritonavir extending into the active site while lopinavir as well as ritonavir bound to the allosteric site of 3CLpro. This binding pose could be attributed to their chemical structures requiring a more substantial binding pocket. Chloroquine (S-RBD and ACE2) and hydroxychloroquine (TMPRSS2) were also found to bind to allosteric sites after docking (Figures S6 and S7, respectively). The binding of hydroxychloroquine in the positively charged allosteric site of TMPRSS2 was similar to earlier findings of remdesivir binding with TMPRSS2 where hydrogen bonds with ASN and ARG were also formed [20]. It is of interest that the high binding energies of 3-galloylcatechin, proanthocyanidin B1, and luteolin 7-galactoside to ACE2 and TMPRSS2 could have clinical implication in the prevention of SARS-CoV-2 transmission [66]. Various studies have reported other phytomolecules present in African medicinal plants that have exhibited potential activity as antagonists against SARS-CoV-2 by interfering with different therapeutic targets [67, 68]. These findings further buttress the role of NPs and their identified bioactives in tackling the COVID-19 pandemic.

### 3.3. Physicochemical, Pharmacokinetic, and Toxicity Evaluation of Hit Ligands

The physicochemical properties of the hit ligands are presented in Table 3. Interestingly, none of the hit molecules were able to pass Lipinski's druglikeness test with 3-galloylcatechin, proanthocyanidin B1, and luteolin 7-galactoside violating 1, 3, and 2 rules, respectively. They also violated Ghose, Veber, Egan, Muegge, and leadlikeness parameters except for 3-galloylcatechin and luteolin 7-galactoside, which passed Ghose filters. Synthetic availability score (4.16, 5.32, and 5.17, respectively) and 10% bioavailability score (0.55, 0.17, and 0.17, respectively) were predicted for 3-galloylcatechin, proanthocyanidin B1, and luteolin 7-galactoside. Lipinski's rule of 5 and its variants are used to predict if small chemical compounds detected as pharmaceutical leads are orally active [69–72]. 3-Galloylcatechin could be ascertained to be orally active based on a consensus that only one parameter is violated in Lipinski's RO5 [36]. The druglikeness failure exhibited by the compounds is not surprising as NPs have been reported to fail Lipinski's druglikeness test, which is attributed to their mechanism of absorption [73]. NPs have been reported to be bioavailable by exploiting complex mechanisms such as active transport unlike synthetic drugs that utilise passive diffusion [74]. This is because NPs look a lot like biosynthetic intermediates and endogenous metabolites than synthetic compounds [75]. They also differ in terms of elemental composition and stereochemical complexity [76]. Interestingly, many NPs that violate RO5 have been reported to remain bioavailable by Lipinski [77]. Advances in synthetic biology and organic synthesis methodology such as biosynthetic gene cluster manipulation, total synthesis, semisynthesis, or a combination of these methods have identified a new generation of natural product scaffolds that can be systematically targeted, to increase the activity, decrease the toxicity, and/or improve the physicochemical and pharmacokinetic properties [78]. This can also be applied as done in other natural leads during synthesis and lead optimization to improve the druglikeness of the compounds [79, 80]. The synthetic accessibility score of the compounds ranged from 4.16 to 5.32. The score which ranges from 1 to 10 is based primarily on the assumption that molecular fragment frequency in easily obtainable molecules actually correlate with the ease of synthesis [44]. The observed values for the hit ligands suggest their fragmental contribution and chemical moieties should be moderately favourable for synthetic synthesis in the pharmaceutical industry leading to potential drug discovery outcome [81–83].

In the course of drug discovery, compounds with predicted favourable pharmacokinetic and toxicity properties have the potential to pass standard clinical trial criteria's making them drug candidates [84]. Looking at the absorption properties presented in Table 4, none of the hit compounds were permeable to Caco-2 and human intestine. They were also not P-glycoprotein substrates, inhibitors, and bioavailable at 30%. However, 3-galloylcatechin was predicted to inhibit the P-glycoprotein, permeate the blood-brain barrier, and become bioavailable at 20%. In contrast, proanthocyanidin B1 and luteolin 7-galactoside could permeate the blood-brain barrier and become bioavailable at 20%, respectively. These compounds are not P-glycoprotein substrates, ensuring their bioavailability and intracellular concentration are not affected [85]. A look at the distribution properties showed 87.287, 76.369, and 78.270% as the plasma protein binding and −1.129, −0.720, and −1.028 L/kg as the volume distribution for 3-galloylcatechin, proanthocyanidin B1, and luteolin 7-galactoside, respectively. These hit compounds were predicted to be neither inhibitors nor substrate of the various cytochrome P_450_ (CYP_450_) isoforms. 3-Galloylcatechin was nonetheless the only exception inhibiting CYP3A4 and CYP2C19 isoforms (Table 4). This inhibition could lead to the accumulation of drugs metabolised by these CYP isoforms and, hence, drug toxicity may occur [86]. A half-life of 1.534, 2.11, and 1.483 hours as well as a clearance rate of 1.204, 1.015, and 1.232 mL/min/kg were the respective elimination properties of 3-galloylcatechin, proanthocyanidin B1, and luteolin 7-galactoside (Table 4). Toxicity property presented in Table 4 showed these compounds met the maximum recommended daily dose by the U.S. Food and Drug Administration without being hepatotoxic, skin sensitisers, and mutagenic. They were found to be human ether-a-go-go-related (hERG) gene blocker with the ability to induce drug liver injury. On the other hand, 3-galloylcatechin was found to be mutagenic, while luteolin 7-galactoside was not a predicted hERG-related gene blocker. Compounds identified as inhibitors of CYP isoforms, hERG blockers, and AMES mutagen can be optimised by the addition of analogues to their cores during optimization process to avoid the development of long QT syndrome and mutagenicity and overcome the few lapses in the pharmacokinetic properties [78]. These identified hits, however, possess good ADMET properties while meeting the maximum recommended daily dose of USFDA.

## 4. Conclusion

In this study, computer-aided analysis was utilised to identify 3-galloylcatechin, proanthocyanidin B1, and luteolin 7-galactoside (ZINC 3978503, ZINC 5085289, and ZINC 40422816, respectively) found in some medicinal plants (almond (*Terminalia catappa*), grape (*Vitis vinifera*), and common verbena (*Verbena officinalis*)) of African flora as hit compounds against multiple SARS-CoV-2 targets. These compounds could be possible leads and nutraceuticals involved in the treatment or as prophylaxis of COVID-19. The scaffolds of these compounds can be optimised to improve the few lapses in its metabolism and toxicity. The results further suggest these compounds will help to overcome in some degree the old paradigm “one gene, one drug, one disease” of drug discovery. Nonetheless, further in vitro, in vivo, and clinical research is required to validate the pharmacotherapeutic significance of these hit compounds as anti-SARS-CoV-2 therapy.

## Figures and Tables

**Table 1:** Grid box coordinates and size parameters used for molecular docking.

	PLpro	3CLpro	RdRp	Helicase	2OMT	S-RBD	ACE2	TMPRSS2
Dimension (Å)								
*x*	20	18	28	28	22	25	60	34
*y*	24	34	26	24	23	20	42	40
*z*	26	30	32	32	23	25	26	34

Centre (Å)								
*x*	−35	−20	115	415	90	175	180	9
*y*	30	25	115	−11	15	115	107	−1
*z*	33	65	127	85	25	250	255	19

PLpro: papain-like protease; 3CLpro: main/3-chymotrypsin-like protease; RdRp: RNA-dependent RNA polymerase; 2OMT: 2-O-methyltransferase; S-RBD: spike receptor-binding domain; ACE2: human angiotensin-converting enzyme 2; TMPRSS2: human type-II transmembrane serine protease.

**Table 2:** Docking results of ligands and standard drugs on SARS-CoV-2 therapeutic targets.

	PLpro	3CLpro	Helicase	RdRp	2OMT	S-RBD	ACE2	TMPRSS2
Binding energy (kcal/mol)								
3-Galloylcatechin	**−5.7**	**−6.3**	−9.8	−8.7	−8.6	−5.2	−8.5	−8.4
Proanthocyanidin B1	−4.1	−5.1	**−10.3**	**−9.8**	**−10.5**	**−6.4**	**−9.7**	**−8.9**
Luteolin 7-galactoside	−4.6	−5.6	−9.2	−8.5	−9.6	−6.1	−8.9	**−8.9**
Camostat mesylate	—	—	—	—	—	—	—	−7
Chloroquine	−4.7	−4.6	−6.4	−4.9	−6.1	−4.9	−5.1	−6
Hydroxychloroquine	−4.6	−5.3	−6.5	−5.5	−6.3	−4	−5.7	−6.2
Lopinavir	−3.7	−4.1	−8.2	−7.9	−6.9	−5.1	−7.1	—
Nafamostat mesylate	—	—	—	—	—	—	—	−7.9
Remdesivir	−3.6	−5.2	−8.1	−7.3	−7.2	−5.5	−8.1	−7.1
Ritonavir	−4.2	−4.1	−8.4	−6.3	−6.9	−4.5	−7.9	—

The values in bold represent the best scores for each target. PLpro: papain-like protease; 3CLpro: main/3-chymotrypsin-like protease; RdRp: RNA-dependent RNA polymerase; 2OMT: 2-O-methyltransferase; S-RBD: spike receptor-binding domain; ACE2: human angiotensin-converting enzyme 2; TMPRSS2: human type-II transmembrane serine protease.

**Table 3:** Predicted physicochemical and druglikeness properties of potential SARS-CoV-2 multitarget hit ligands.

	3-Galloylcatechin	Proanthocyanidin B1	Luteolin 7-galactoside
Molecular weight (g/mol)	442.37	578.52	448.38
Hydrogen bond donor	7	10	7
Hydrogen bond acceptor	10	12	11
LogP	0.05	−0.26	−2.10
LogS	−3.7	−5.14	−3.65
Topological polar surface area	177.14	220.76	190.28
#Rotatable bonds	4	3	4
Molar refractivity	110.04	146.71	108.13
Bioavailability (10%)	0.55	0.17	0.17
Synthetic accessibility	4.16	5.32	5.17
#Heavy atoms	32	42	32
#Lipinski violation	1	3	2
#Ghose violation	0	2	0
#Veber violation	1	1	1
#Egan violation	1	1	1
#Muegge violation	2	3	3
#Leadlikeness violation	1	1	1

LogP: molecular lipophilicity; LogS: aqueous solubility.

**Table 4:** Predicted ADMET properties of potential SARS-CoV-2 multitarget hit compounds.

	3-Galloylcatechin	Proanthocyanidin B1	Luteolin 7-galactoside
Absorption			
Caco-2 permeability (cm/s)	−(−6.782)	−(−6.782)	−(−6.393)
Pgp-inhibitor	++	—	—
Pgp-substrate	—	—	—
Blood-brain barrier	++	+	—
Human intestinal absorption	—	—	—
*F*_20%_	+	—	+
*F*_30%_	—	—	—

Distribution			
Plasma protein binding (%)	87.287	76.369	78.270
Volume distribution (L/kg)	−1.129	−0.720	−1.028

Metabolism			
CYP1A2 inhibitor	—	—	—
CYP1A2 substrate	—	—	—
CYP3A4 inhibitor	+	—	—
CYP3A4 substrate	—	—	—
CYP2C9 inhibitor	—	—	—
CYP2C9 substrate	—	—	—
CYP2C19 inhibitor	+	—	—
CYP2C19 substrate	—	—	—
CYP2D6 inhibitor	—	—	—
CYP2D6 substrate	—	—	—

Elimination			
*T*_1/2_ (h)	1.534	2.11	1.483
Clearance rate (mL/min/kg)	1.204	1.015	1.232

Toxicity			
hERG blocker	+	+	—
Human hepatotoxicity	—	—	—
AMES mutagenicity	+	—	—
Skin sensitisation	—	—	—
LD_50_ (mg/kg)	538.011	357.539	418.453
Drug-induced liver injury	++	++	+
FDAMDD	+	++	++

Pgp, *F*_20/30%_, *T*_1/2_, hERG, and FDAMDD represent P-glycoprotein, bioavailability, half-life time, human ether-a-go-go-related gene, and U.S. Food and Drug Administration Maximum Recommended Daily Dose, respectively. “—,” “−,” “+,” “++,” and “+++” signify the level of the predicted property.

## Data Availability

The data used to support the findings of this study are included within the article and supplementary files.

## Supplementary Materials

Supplementary Materials Table S1: list of molecules downloaded from ZINC database subset (AfroDb Natural Products). Table S2: homology modelling result for SARS-CoV-2 helicase. Table S3: verification of stereochemical quality of SARS-CoV-2 helicase template and modelled and minimised modelled structure. Table S4: virtual screening result of molecules against multiple SARS-CoV-2 targets using iGEMDOCK. Figure S1: helicase homology model-template sequence alignment. Figure S2: 3D crystal structure of (a) homology modelled SARS-CoV-2 and (b) structural superimposition of 5wwp (blue), modelled helicase (white), and energy minimised modelled helicase (green). Figure S3: 3D verification plot of the minimised modelled SARS-CoV-2 helicase structure. Figure S4: quality factor plot of the minimised modelled SARS-CoV-2 helicase structure. Figure S5: predicted binding pockets of (a) PLpro, (b) 3CLpro, (c) helicase, (d) RdRp, (e) 2OMT, (f) S-RBD, and (g) ACE2 and TMPRSS2 by DogSiteScorer. Figure S6: 3D representation of ZINC 3978503, ZINC 5085289, ZINC 40422816, chloroquine, hydroxychloroquine, lopinavir, remdesivir, and ritonavir colour coded as red, blue, green, yellow, purple, black, orange, and magenta, respectively, in the binding pocket of (a) PLpro, (b) 3CLpro, (c) helicase, (d) RdRp, (e) 2OMT, (f) S-RBD, and (g) ACE2. Figure S7: 3D representation of ZINC 3978503, ZINC 5085289, ZINC 40422816, camostat, chloroquine, hydroxychloroquine, and nafamostat in the binding pocket of TMPRSS2 colour coded as red, blue, green, yellow, black, orange, and pink, respectively.
