# Supplementary material for: Computer-Aided Analysis of Multiple SARS-CoV-2 Therapeutic Targets: Identification of Potent Molecules from African Medicinal Plants
Source: Scientifica (Cairo). 2020 Sep 12;2020:1878410. doi: 10.1155/2020/1878410 (PMC7492903; doi:10.1155/2020/1878410)
Supplement: Supplementary Materials — Table S1: list of molecules downloaded from ZINC database subset (AfroDb Natural Products). Table S2: homology modelling result for SARS-CoV-2 helicase. Table S3: verification of stereochemical quality of SARS-CoV-2 helicase template and modelled and minimised modelled structure. Table S4: virtual screening result of molecules against multiple SARS-CoV-2 targets using iGEMDOCK. Figure S1: helicase homology model-template sequence alignment. Figure S2: 3D crystal structure of (a) homology modelled SARS-CoV-2 and (b) structural superimposition of 5wwp (blue), modelled helicase (white), and energy minimised modelled helicase (green). Figure S3: 3D verification plot of the minimised modelled SARS-CoV-2 helicase structure. Figure S4: quality factor plot of the minimised modelled SARS-CoV-2 helicase structure. Figure S5: predicted binding pockets of (a) PLpro, (b) 3CLpro, (c) helicase, (d) RdRp, (e) 2OMT, (f) S-RBD, and (g) ACE2 and TMPRSS2 by DogSiteScorer. Figure S6: 3D representation of ZINC 3978503, ZINC 5085289, ZINC 40422816, chloroquine, hydroxychloroquine, lopinavir, remdesivir, and ritonavir colour coded as red, blue, green, yellow, purple, black, orange, and magenta, respectively, in the binding pocket of (a) PLpro, (b) 3CLpro, (c) helicase, (d) RdRp, (e) 2OMT, (f) S-RBD, and (g) ACE2. Figure S7: 3D representation of ZINC 3978503, ZINC 5085289, ZINC 40422816, camostat, chloroquine, hydroxychloroquine, and nafamostat in the binding pocket of TMPRSS2 colour coded as red, blue, green, yellow, black, orange, and pink, respectively. [file 1878410.f1.zip › 1878410.f1/S-figures Computer aided ligand based screening for identification of promising molecules.docx]

**Computer aided analysis of multiple SARS-CoV-2 therapeutic targets: Identification of potent molecules from African medicinal plants**

Franklyn Nonso Iheagwam^1,2,*^ and Solomon Oladapo Rotimi^1,*^

^1^Department of Biochemistry, College of Science and Technology, Covenant University, Canaanland, P.M.B. 1023, Ota, Ogun State, Nigeria.

^2^Covenant University Public Health and Wellness Research Cluster (CUPHWERC), College of Science and Technology, Covenant University, Canaanland, P.M.B. 1023, Ota, Ogun State, Nigeria

*Corresponding author: [franklyn.iheagwam@covenantuniversity.edu.ng](mailto:franklyn.iheagwam@covenantuniversity.edu.ng) and [ola.rotimi@covenantuniversity.edu.ng](mailto:ola.rotimi@covenantuniversity.edu.ng)

CoV-2 helicase --AVGACVLCNSQTSLRCGACIRRPFLCCKCCYDHVISTSHKLVLSVNPYVCNAPGCDVT

5WWP_1|Chains GPAVGSCVVCHSQTSLRCGTCIRRPFLCCKCCYDHVIATPHKMVLSVSPYVCNAPGCGVS

***:**:*:********:*****************:*.**:****.*********.*:

CoV-2 helicase DVTQLYLGGMSYYCKSHKPPISFPLCANGQVFGLYKNTCVGSDNVTDFNAIATCDWTNAG

5WWP_1|Chains DVTKLYLGGMSYFCVDHRPVCSFPLCANGLVFGLYKNMCTGSPSIVEFNRLATCDWTESG

***:********:* .*:* ******** ******* *.** .:.:** :******::*

CoV-2 helicase DYILANTCTERLKLFAAETLKATEETFKLSYGIATVREVLSDRELHLSWEVGKPRPPLNR

5WWP_1|Chains DYTLANTTTEPLKLFAAETLRATEEASKQSYAIATIKEIVGERQLLLVWEAGKSKPPLNR

** **** ** *********:****: * **.***::*::.:*:* * **.**.:*****

CoV-2 helicase NYVFTGYRVTKNSKVQIGEYTFEKGDYGDAVVYRGTTTYKLNVGDYFVLTSHTVMPLSAP

5WWP_1|Chains NYVFTGYHITKNSKVQLGEYIFERIDYSDAVSYKSSTTYKLTVGDIFVLTSHSVATLTAP

*******::*******:*** **: **.*** *:.:*****.*** ******:* .*:**

CoV-2 helicase TLVPQEHYVRITGLYPTLNISDEFSSNVANYQKVGMQKYSTLQGPPGTGKSHFAIGLALY

5WWP_1|Chains TIVNQERYVKITGLYPTITVPEEFASHVANFQKSGYSKYVTVQGPPGTGKSHFAIGLAIY

*:* **:**:*******:.:.:**:*:***:** * .** *:****************:*

CoV-2 helicase YPSARIVYTACSHAAVDALCEKALKYLPIDKCSRIIPARARVECFDKFKVNSTLEQYVFC

5WWP_1|Chains YPTARVVYTACSHAAVDALCEKAFKYLNIAKCSRIIPAKARVECYDRFKVNETNSQYLFS

**:**:*****************:*** * ********:*****:*:****.* .**:*.

CoV-2 helicase TVNALPETTADIVVFDEISMATNYDLSVVNARLRAKHYVYIGDPAQLPAPRTLLTKGTLE

5WWP_1|Chains TINALPETSADILVVDEVSMCTNYDLSIINARIKAKHIVYVGDPAQLPAPRTLLTRGTLE

*:******:***:*.**:**.******::***::*** **:**************:****

CoV-2 helicase PEYFNSVCRLMKTIGPDMFLGTCRRCPAEIVDTVSALVYDNKLKAHKDKSAQCFKMFYKG

5WWP_1|Chains PENFNSVTRLMCNLGPDIFLSMCYRCPKEIVSTVSALVYNNKLLAKKELSGQCFKILYKG

** **** *** .:***:**. * *** ***.*******:*** *:*: *.****::***

CoV-2 helicase VITHDVSSAINRPQIGVVREFLTRNPAWRKAVFISPYNSQNAVASKILGLPTQTVDSSQG

5WWP_1|Chains NVTHDASSAINRPQLTFVKNFITANPAWSKAVFISPYNSQNAVSRSMLGLTTQTVDSSQG

:***.********: .*::*:* **** **************: .:***.*********

CoV-2 helicase SEYDYVIFTQTTETAHSCNVNRFNVAITRAKVGILCIMSDRDLYDKLQFTSLEIPRRNVA

5WWP_1|Chains SEYQYVIFCQTADTAHANNINRFNVAITRAQKGILCVMTSQALFESLEFTELSFTNYKLQ

***:**** **::***: *:**********: ****:*:.: *::.*:**.*.:.. ::

CoV-2 helicase TLQ

5WWP_1|Chains ---

**Figure S1: Helicase homology model-template sequence alignment**.

| a) 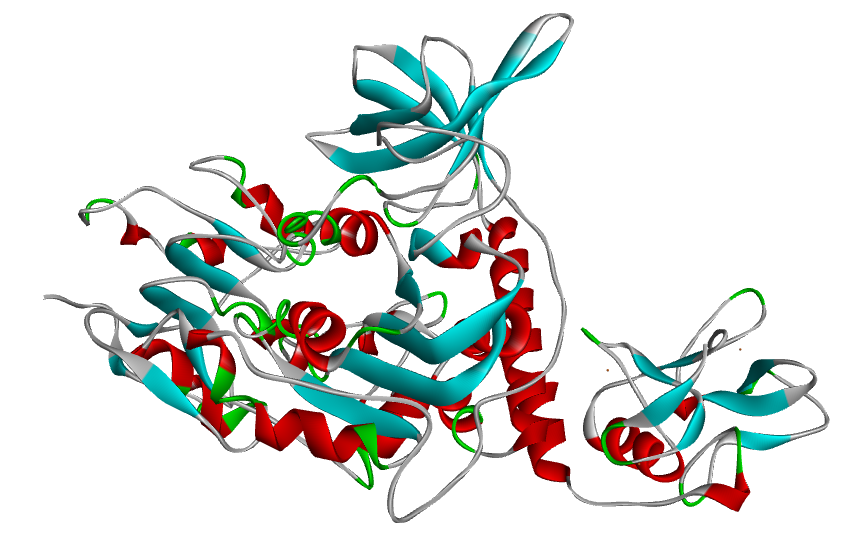 | b)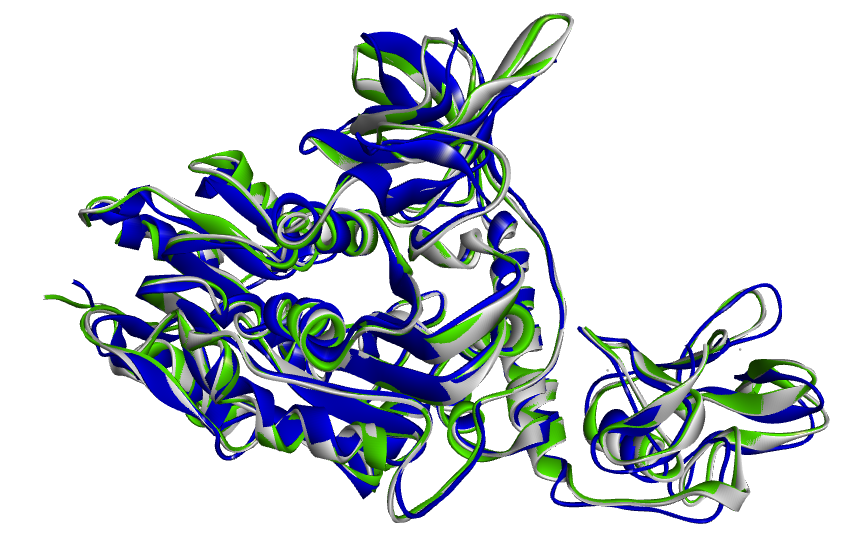 |
| --- | --- |

**Figure S2: 3D crystal structure of (a) homology modelled SARS-CoV-2 and (b) structural superimposition of 5wwp (blue), modelled helicase (white) and energy minimized modelled helicase (green).**


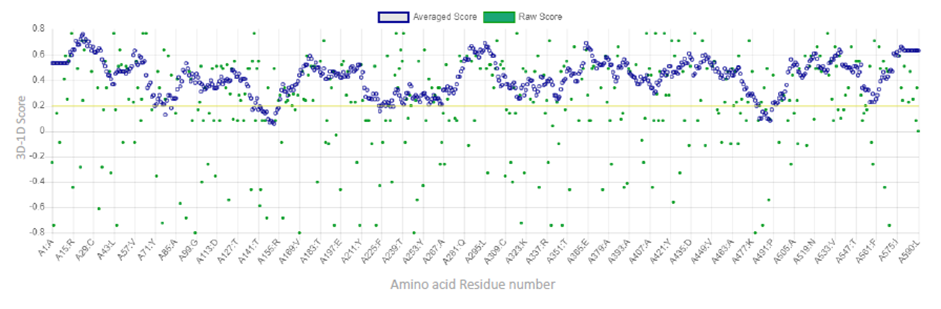


**Figure S3: 3D verification plot of the minimized modelled SARS-CoV-2 helicase structure**


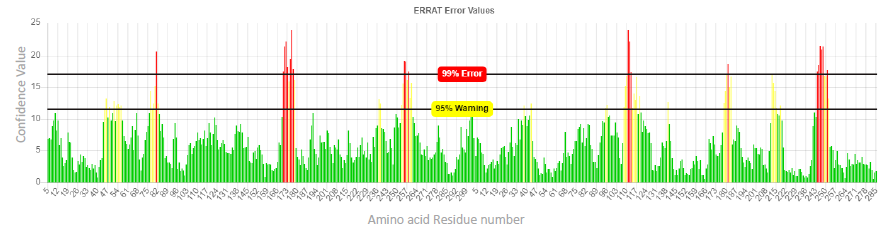


**Figure S4: Quality factor plot of the minimised modelled SARS-CoV-2 helicase structure**

| a) **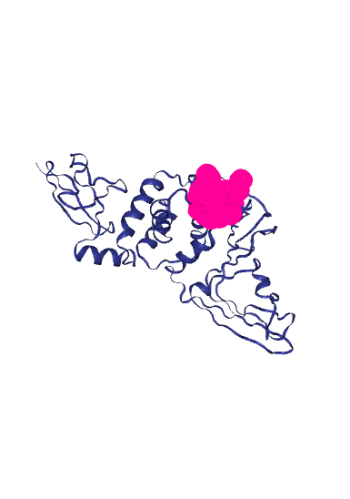** | b) **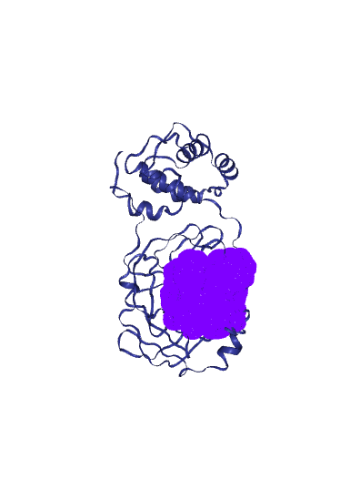** |
| --- | --- |
| c) **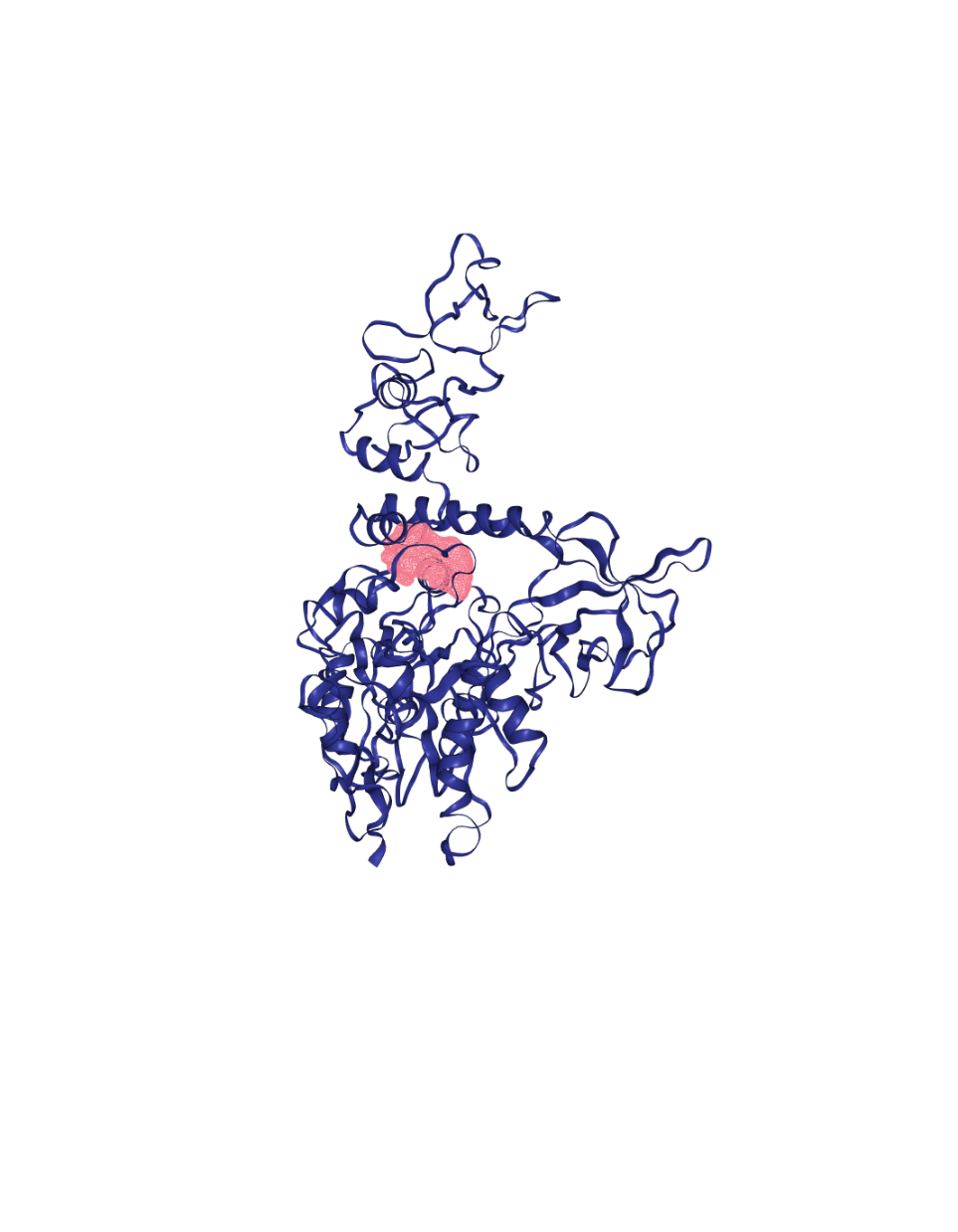** | d) **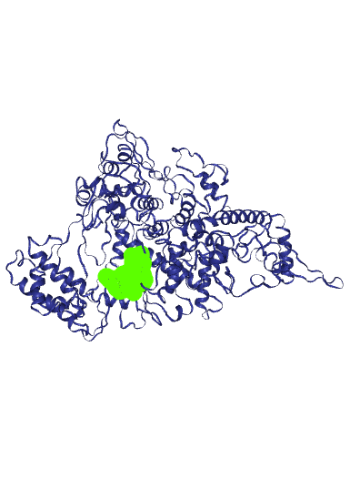** |
| e) **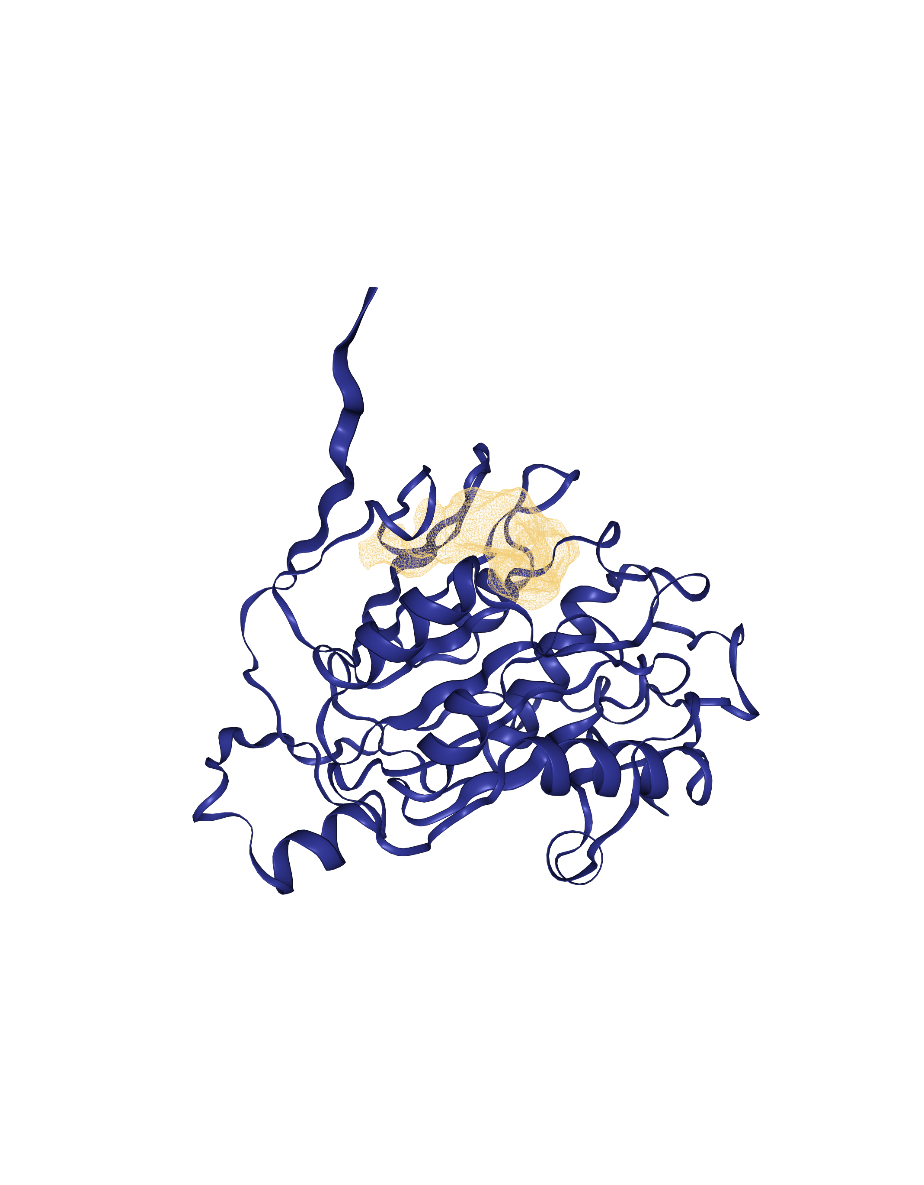** | f) **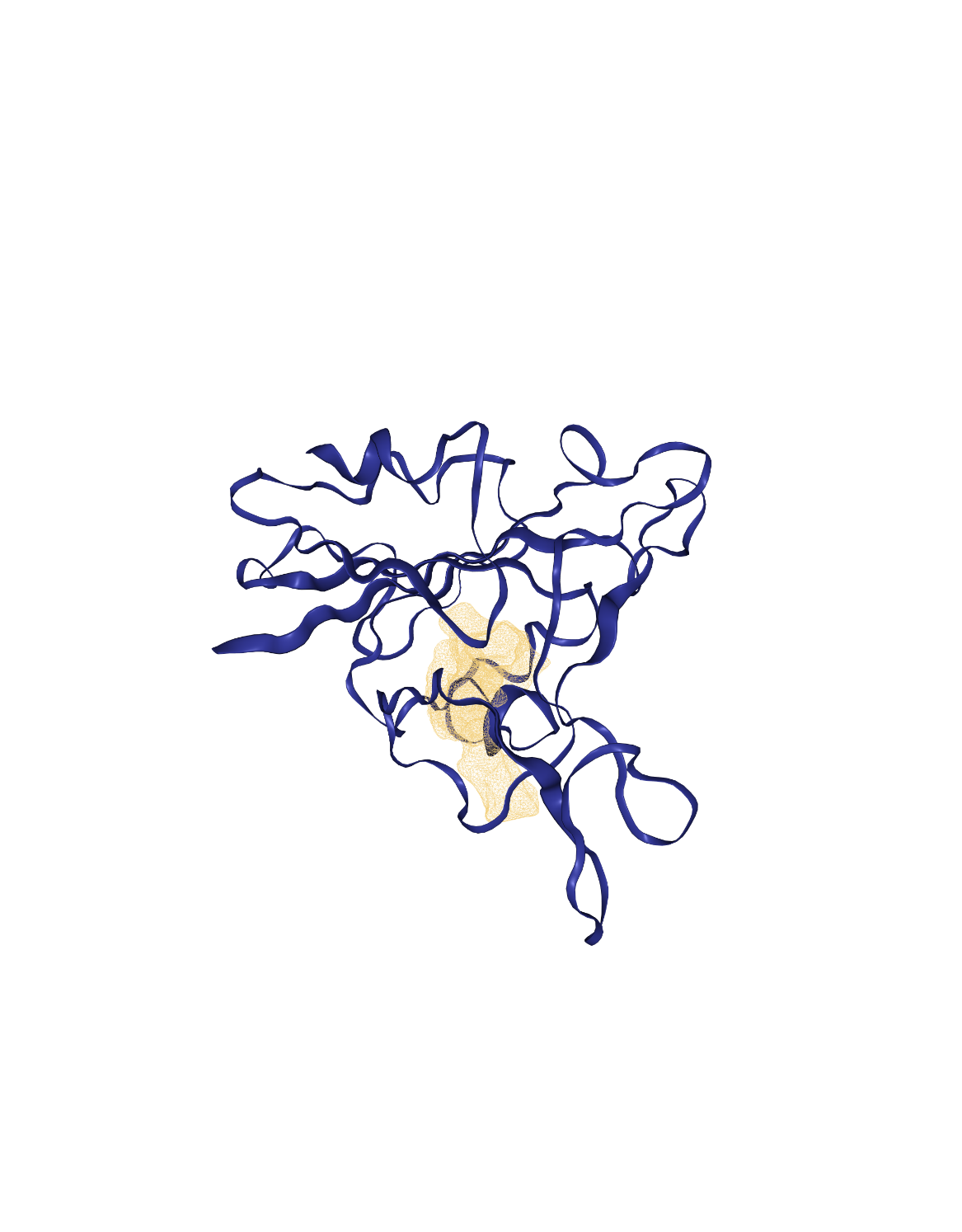** |
| g) **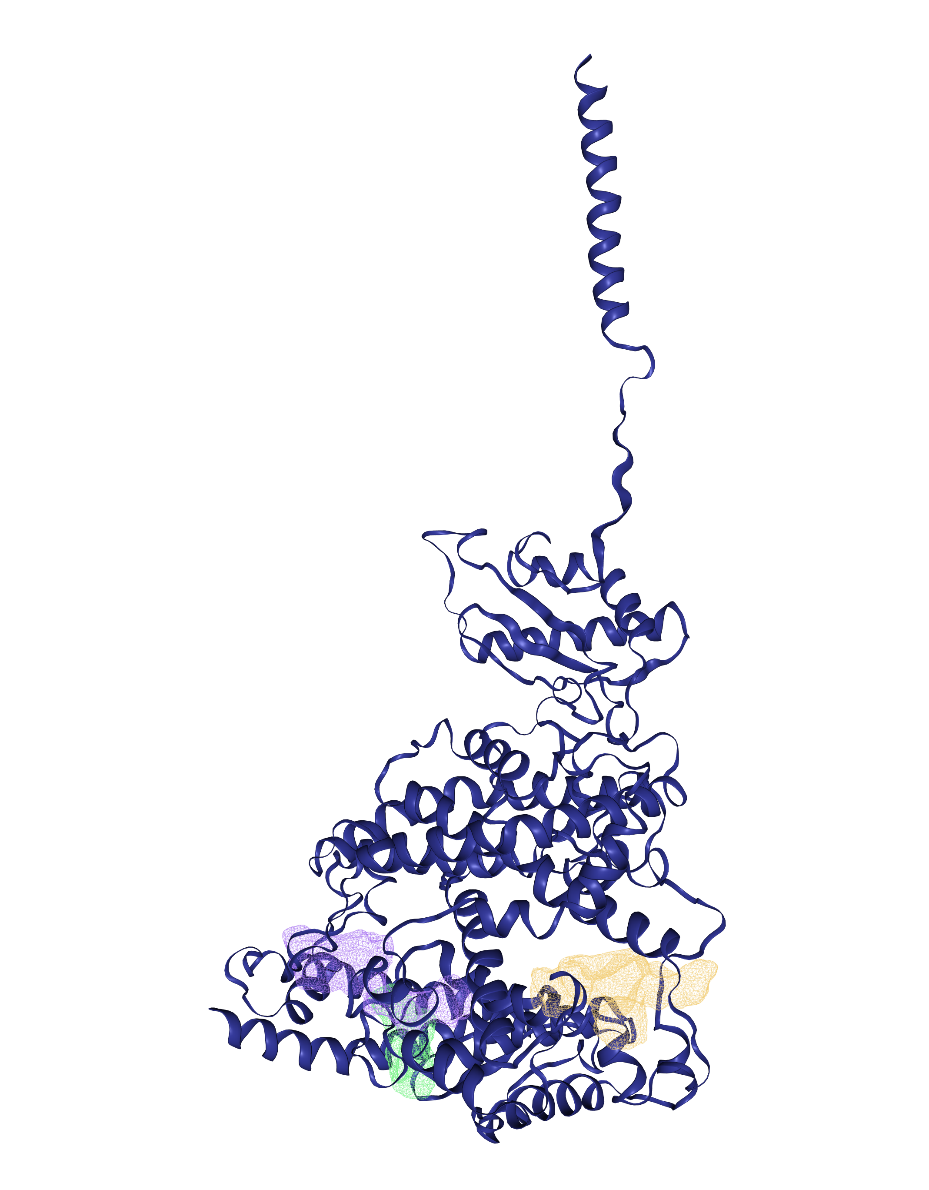** | h) **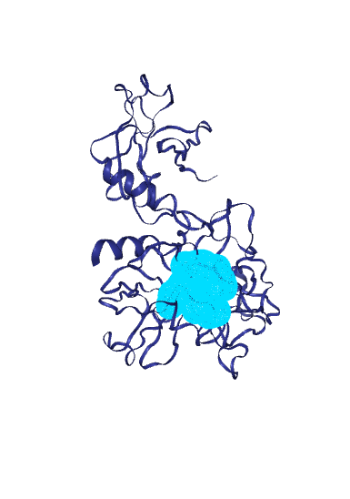** |

**Figure S5: Predicted binding pockets of (a) PLpro (b) 3CLpro (c) helicase (d) RdRp (e) 2OMT (f) S-RBD (g) ACE2 and TMPRSS2 by DogSiteScorer**

a)
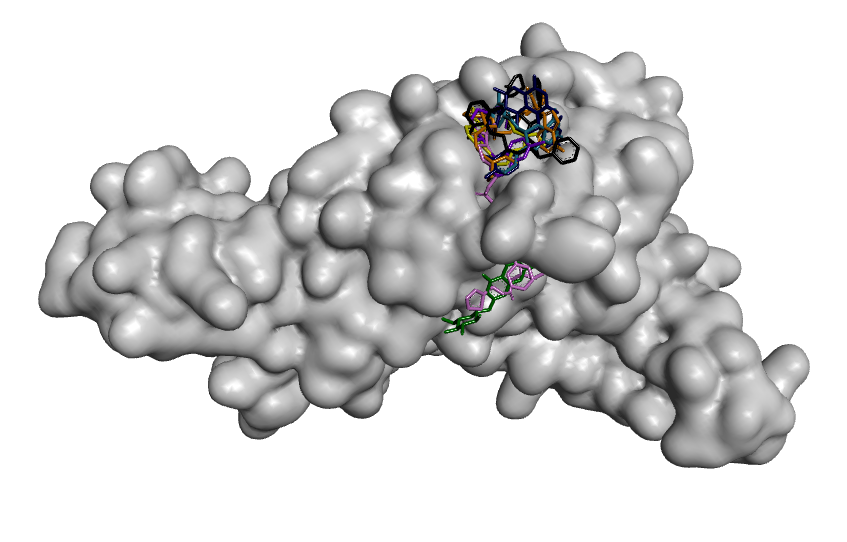


b)
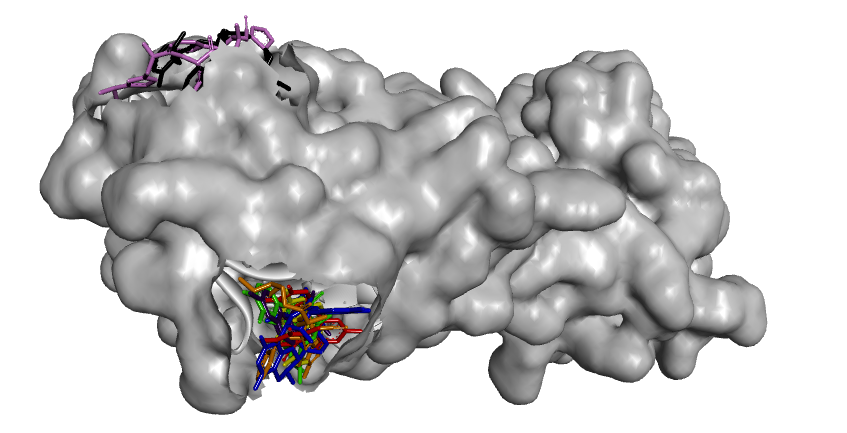


c)
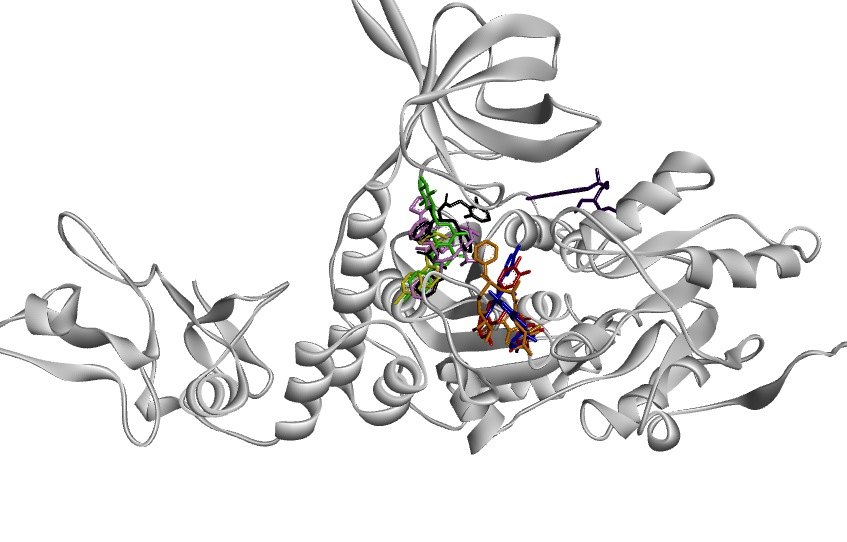


d)
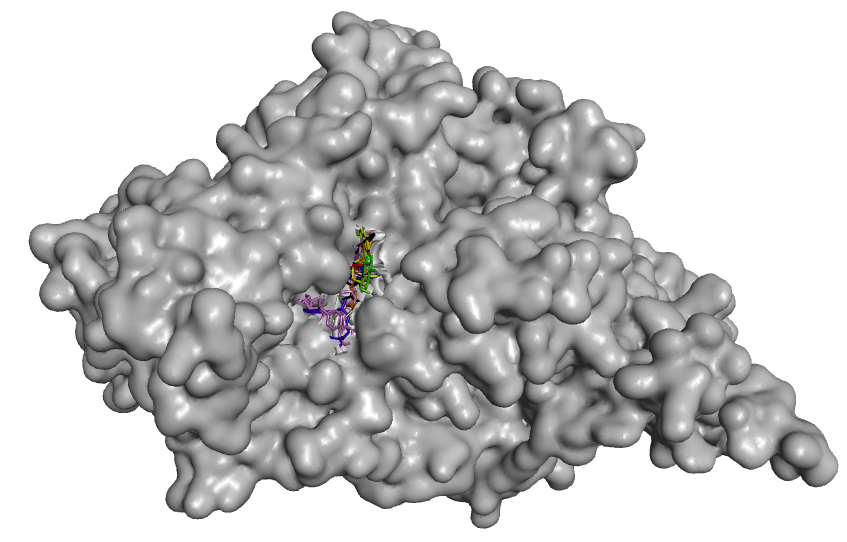


e)
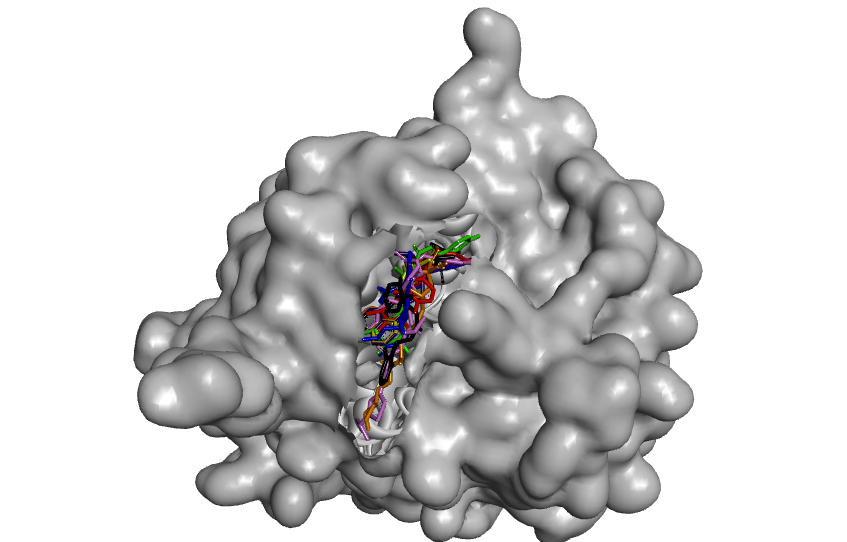


f)
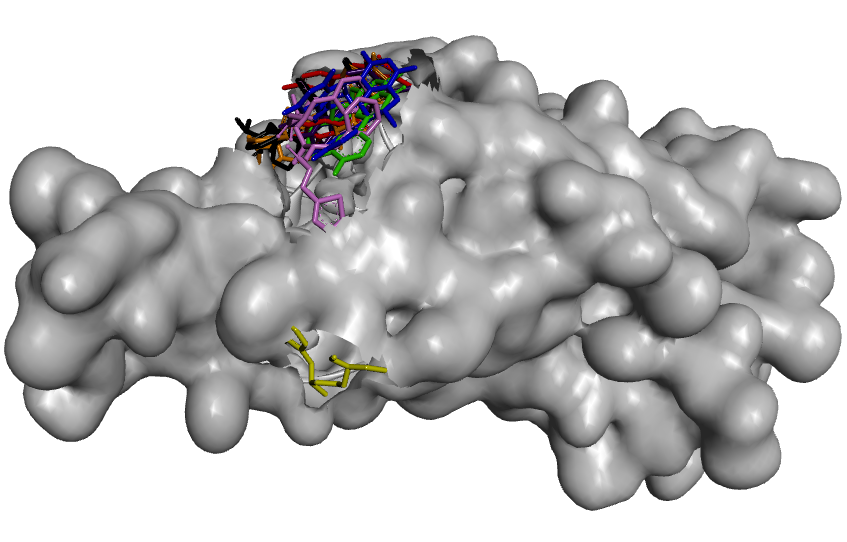


g)
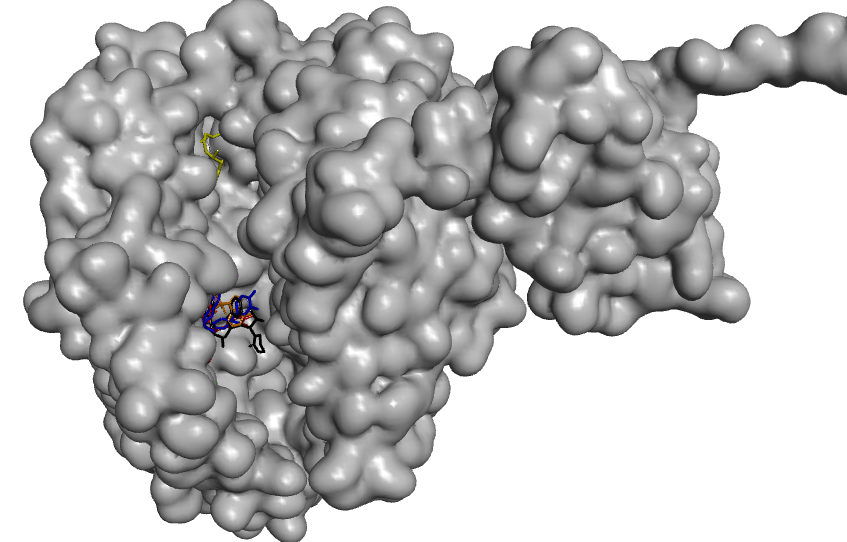


**Figure S6: 3D representation of ZINC 3978503, ZINC 5085289, ZINC 40422816, chloroquine, hydroxychloroquine, lopinavir, remdesivir and ritonavir colour coded as red, blue, green, yellow, purple, black, orange and magenta respectively in the binding pocket of (a) PLpro (b) 3CLpro (c) helicase (d) RdRp (e) 2OMT (f) S-RBD and (g) ACE2**


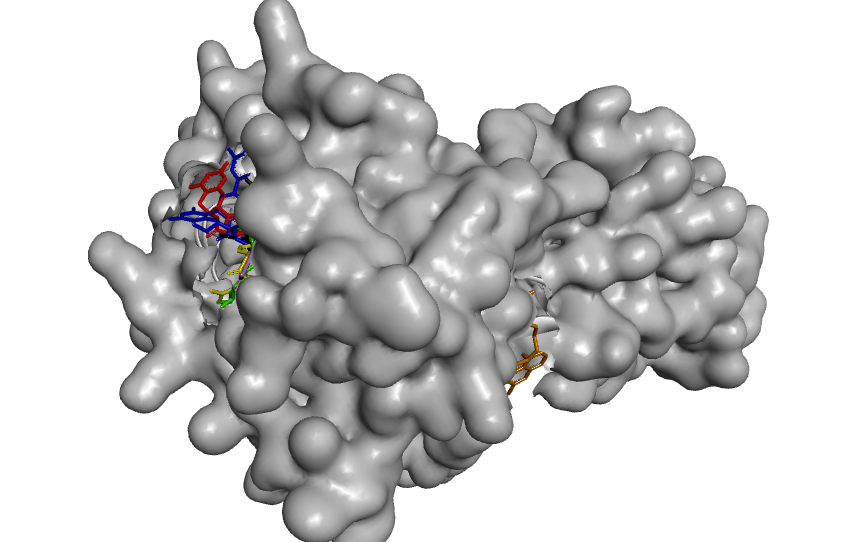


**Figure S7: 3D representation of ZINC 3978503, ZINC 5085289, ZINC 40422816, camostat, chloroquine, hydroxychloroquine and nafamostat in the binding pocket of TMPRSS2 colour coded as red, blue, green, yellow, black, orange and pink respectively.**
