# Supplementary material for: Computer-Aided Analysis of Multiple SARS-CoV-2 Therapeutic Targets: Identification of Potent Molecules from African Medicinal Plants
Source: Scientifica (Cairo). 2020 Sep 12;2020:1878410. doi: 10.1155/2020/1878410 (PMC7492903; doi:10.1155/2020/1878410)
Supplement: Supplementary Materials — Table S1: list of molecules downloaded from ZINC database subset (AfroDb Natural Products). Table S2: homology modelling result for SARS-CoV-2 helicase. Table S3: verification of stereochemical quality of SARS-CoV-2 helicase template and modelled and minimised modelled structure. Table S4: virtual screening result of molecules against multiple SARS-CoV-2 targets using iGEMDOCK. Figure S1: helicase homology model-template sequence alignment. Figure S2: 3D crystal structure of (a) homology modelled SARS-CoV-2 and (b) structural superimposition of 5wwp (blue), modelled helicase (white), and energy minimised modelled helicase (green). Figure S3: 3D verification plot of the minimised modelled SARS-CoV-2 helicase structure. Figure S4: quality factor plot of the minimised modelled SARS-CoV-2 helicase structure. Figure S5: predicted binding pockets of (a) PLpro, (b) 3CLpro, (c) helicase, (d) RdRp, (e) 2OMT, (f) S-RBD, and (g) ACE2 and TMPRSS2 by DogSiteScorer. Figure S6: 3D representation of ZINC 3978503, ZINC 5085289, ZINC 40422816, chloroquine, hydroxychloroquine, lopinavir, remdesivir, and ritonavir colour coded as red, blue, green, yellow, purple, black, orange, and magenta, respectively, in the binding pocket of (a) PLpro, (b) 3CLpro, (c) helicase, (d) RdRp, (e) 2OMT, (f) S-RBD, and (g) ACE2. Figure S7: 3D representation of ZINC 3978503, ZINC 5085289, ZINC 40422816, camostat, chloroquine, hydroxychloroquine, and nafamostat in the binding pocket of TMPRSS2 colour coded as red, blue, green, yellow, black, orange, and pink, respectively. [file 1878410.f1.zip › 1878410.f1/S-tables Computer aided ligand based screening for identification of promising molecules.docx]

**Computer aided analysis of multiple SARS-CoV-2 therapeutic targets: Identification of potent molecules from African medicinal plants**

Franklyn Nonso Iheagwam^1,2,*^ and Solomon Oladapo Rotimi^1,*^

^1^Department of Biochemistry, College of Science and Technology, Covenant University, Canaanland, P.M.B. 1023, Ota, Ogun State, Nigeria.

^2^Covenant University Public Health and Wellness Research Cluster (CUPHWERC), College of Science and Technology, Covenant University, Canaanland, P.M.B. 1023, Ota, Ogun State, Nigeria

*Corresponding author: [franklyn.iheagwam@covenantuniversity.edu.ng](mailto:franklyn.iheagwam@covenantuniversity.edu.ng) and [ola.rotimi@covenantuniversity.edu.ng](mailto:ola.rotimi@covenantuniversity.edu.ng)

**Table S1: List of molecules downloaded from ZINC database subset (AfroDb Natural Products)**

| PDB No. | ZINC ID | CANONICAL SMILES | COMPOUND NAME |
| --- | --- | --- | --- |
|  | ZINC1411 | C=CCc1ccc(O)c(OC)c1 | Eugenol |
|  | ZINC1504 | O=C(O)c1cc(O)c(O)c(O)c1 | Galop |
|  | ZINC2054 | OCc1ccccc1O | Saligenin |
|  | ZINC13245 | O=Cc1ccc(O)c(O)c1 | Protocatechualdehyde |
|  | ZINC13246 | O=C(O)c1ccc(O)c(O)c1 | B-Resorcylate |
|  | ZINC39811 | O=C(O)/C=C/c1ccc(O)cc1 | P-cumarate |
|  | ZINC56792 | COc1ccc2c(c1O)-c1c(OC)c(OC)cc3c1[C@H](C2)N(C)CC3 | isocorydine |
|  | ZINC57731 | COc1c2ccoc2cc2oc(=O)ccc12 | 5-Mop |
|  | ZINC57733 | COc1cc2ccc(=O)oc2cc1O | Buxuletin |
|  | ZINC119983 | Oc1cc(O)c2c(c1)O[C@H](c1ccc(O)c(O)c1)[C@@H](O)C2 | (+/-)-Catechin |
|  | ZINC119988 | Oc1cc(O)c2c(c1)O[C@H](c1ccc(O)c(O)c1)[C@H](O)C2 | Catechin |
|  | ZINC120283 | O=c1ccc2cc3ccoc3cc2o1 | Ficusin |
|  | ZINC135449 | COc1cc2c(cc1O)C[C@H]1c3c(cc(O)c(OC)c3-2)CCN1C | Boldine |
|  | ZINC607790 | COc1cc2c(c(OC)c1OC)-c1ccc(OC)c(=O)cc1[C@H](NC(C)=O)CC2 | Colcrys |
|  | ZINC607911 | COc1cc2c(cc1OC)-c1c(OC)c(OC)cc3c1[C@H](C2)N(C)CC3 | Glaucine |
|  | ZINC1529208 | CC(C)=CCC/C(C)=C/C=O | Citral |
|  | ZINC1530009 | O=c1cc(-c2ccc(O)cc2)oc2cc(O[C@H]3O[C@@H](CO)[C@H](O)[C@@H](O)[C@@H]3O)cc(O)c12 | Apigenin 7-o-β-glucoside |
|  | ZINC1530331 | C=CC(=C)CCC=C(C)C | β-Myrcene |
|  | ZINC1530775 | N=C(N)c1ccc(OCCCCCOc2ccc(C(=N)N)cc2)cc1 | Pentamidine |
|  | ZINC1532860 | CC(C)=CCC/C(C)=C/CC/C(C)=C/CO | Farnesol |
|  | ZINC1535009 | CCN(CC)CCCCCCNc1cc(OC)cc2c(C)ccnc12 | Sitamaquine |
|  | ZINC1597393 | COc1cc2c(cc1-c1cc3cc4ccoc4cc3oc1=O)OCO2 | Neorautone |
|  | ZINC1618678 | CC(C)=CCc1c(O)ccc2c1O[C@H]1c3ccc(O)c(CC=C(C)C)c3OC[C@H]21 | 4-Prenylphaseollidin |
|  | ZINC1697403 | CCCOC(C)=O | Propyl Acetate |
|  | ZINC1721693 | Oc1ccc([C@H]2Oc3cc(O)cc(O)c3C[C@@H]2O)cc1 | Afzelechin |
|  | ZINC2040970 | C=C[C@](C)(O)CC/C=C(\C)CCC=C(C)C | Stirrup |
|  | ZINC3824868 | Cc1cc(O)c2c(c1)C(=O)c1cc(O)cc(O)c1C2=O | Emodin |
|  | ZINC3830747 | CC[C@H]1CN2CCc3cc(OC)c(OC)cc3[C@@H]2C[C@@H]1C[C@H]1NCCc2cc(OC)c(OC)cc21 | Emetine |
|  | ZINC3861087 | C/C(=C\CO)CCC[C@H](C)CCC[C@H](C)CCCC(C)C | e-Phytol |
|  | ZINC3869685 | O=c1c(O)c(-c2ccc(O)c(O)c2)oc2cc(O)cc(O)c12 | Meletin |
|  | ZINC3871576 | O=c1cc(-c2ccc(O)cc2)oc2cc(O)cc(O)c12 | Apigenin |
|  | ZINC3978503 | O=C(O[C@@H]1Cc2c(O)cc(O)cc2O[C@@H]1c1ccc(O)c(O)c1)c1cc(O)c(O)c(O)c1 | 3-Galloylcatechin |
|  | ZINC5085289 | Oc1cc(O)c2c(c1)O[C@H](c1ccc(O)c(O)c1)[C@H](O)[C@@H]2c1c(O)cc(O)c2c1O[C@H](c1ccc(O)c(O)c1)[C@@H](O)C2 | Proanthocyanidin B1 |
|  | ZINC5430812 | COc1c(O)cc2oc3cc(O)c(CC=C(C)C)c(O)c3c(=O)c2c1CC=C(C)C | α-Mangostin |
|  | ZINC5758645 | CC(=O)OC/C=C(\C)CCC[C@H](C)CCC[C@H](C)CCCC(C)C | Phytyl Acetate |
|  | ZINC5783606 | COc1cc2c(c(O)c1OC)c(=O)c1ccccc1n2C | Arborinin |
|  | ZINC5854400 | CC(C)=CCc1c(O)c(CC=C(C)C)c2oc3c(O)ccc(O)c3c(=O)c2c1O | Gartanin |
|  | ZINC6050191 | CC1(C)C=Cc2c(ccc3c2O[C@@H]2c4ccc(O)cc4OC[C@@H]32)O1 | (-)-Phaseollin |
|  | ZINC6523722 | Cn1c2ccccc2c(=O)c2c(O)cccc21 | 1-Hydroxy-N-Methylacridone |
|  | ZINC6524916 | O=c1c2cc(O)ccc2oc2cccc(O)c12 | Purrenone |
|  | ZINC6920379 | CCCCCCCCCCCCCCCCCCCCCCC(=O)O | Tricosanoic acid |
|  | ZINC13282986 | COc1cc2nccc3c4ccccc4n(c1=O)c23 | 5-Methoxycanthinone |
|  | ZINC13382495 | COc1c(O)cc2oc3cc4c(c(O)c3c(=O)c2c1CC=C(C)C)C=CC(C)(C)O4 | Mangostanin |
|  | ZINC13393275 | CCCc1cc(=O)oc2c(C(=O)[C@@H](C)CC)c(O)c(CC=C(C)C)c(O)c12 | (s)-Neomammein |
|  | ZINC13512214 | Oc1ccccc1O | Catechol |
|  | ZINC13539005 | COc1c(O)cc2oc3cc(O)cc(O)c3c(=O)c2c1CC=C(C)C | Dulxanthone D |
|  | ZINC14586037 | CCCc1cc(=O)oc2c(C(=O)CC(C)C)c(O)c(CC=C(C)C)c(O)c12 | Mammein |
|  | ZINC14642643 | COc1cc(C(=O)O[C@@H]2Cc3c(O)cc(O)cc3O[C@@H]2c2ccc(O)c(O)c2)cc(O)c1O | 3-O-(3-Methylgalloyl) epicatechin |
|  | ZINC14689062 | Cn1c2ccccc2c(=O)c2c(O)cc3c(c21)C[C@@H]([C@](C)(O)CO)O3 | Gravacridondiol |
|  | ZINC14768737 | CC(C)=CCc1c(O)c2c(c3c(=O)c4c(O)c5c(cc4oc13)OC(C)(C)C=C5)C=CC(C)(C)O2 | Tovophyllin B |
|  | ZINC14819573 | COc1ccc2c(c1CC=C(C)C)O[C@@H]1c3ccc(O)cc3OC[C@@]21O | Cristacarpin |
|  | ZINC15043216 | O=C(OC[C@H]1O[C@@H](OC(=O)c2cc(O)c(O)c(O)c2)[C@H](O)[C@@H](O)[C@H]1O)c1cc(O)c(O)c(O)c1 | Quinoxaline-1-Oxide |
|  | ZINC15118974 | O=c1c2cccc(O)c2oc2cccc(O)c12 | Calophyllin B |
|  | ZINC18157343 | CC1=CC(=O)C(=C(C)C)CC1 | Piperitenone |
|  | ZINC18185774 | O=c1cc(-c2ccc(O)c(O)c2)oc2cc(O)cc(O)c12 | Luteolin |
|  | ZINC18825330 | O=c1c(-c2ccc(O)cc2)coc2cc(O)cc(O)c12 | Genistein |
|  | ZINC18847044 | COc1cc(O)c2c(=O)c(-c3ccc(O)cc3)coc2c1 | Prunetin |
|  | ZINC18847046 | COc1ccc2c(c1)[nH]c1c(C)nccc12 | Harmine |
|  | ZINC28109109 | COc1cc2c(cc1O)CCN1CC=C3C=C[C@H](OC)C[C@]321 | Erysodine |
|  | ZINC30726889 | COc1cc2c(cc1OC)[C@]13C[C@@H](OC)C=CC1=CCN3CC2 | Erysotrine |
|  | ZINC33831297 | CC1(C)CCC[C@@]2(C)[C@H]1CC[C@@]1(C=O)[C@@H](O)CC(C=O)=CC[C@H]21 | Galanal A |
|  | ZINC38143792 | C[C@@H]1CC[C@]2(C(=O)O)CC[C@]3(C)C(=CC[C@@H]4[C@@]5(C)C[C@@H](O)[C@@H](O)C(C)(C)[C@@H]5CC[C@]43C)[C@@H]2[C@]1(C)O | Euscaphic acid |
|  | ZINC40422816 | O=c1cc(c2ccc(O)c(O)c2)oc2cc(O[C@H]3O[C@@H](CO)[C@H](O)[C@@H](O)[C@@H]3O)cc(O)c12 | Luteolin 7-galactoside |
|  | ZINC53194131 | CO[C@H]1C=CC2=CCN3CCc4cc5c(cc4[C@]23C1)OCO5 | Erythraline |
|  | ZINC95619153 | CC(C)=CCc1c(O)c(C(=O)CC(C)C)c(O)c2c(-c3ccccc3)cc(=O)oc12 | Mammeisin |

**Table S2: Homology modelling result for SARS-CoV-2 helicase**

| **Template** | **Global model quality estimation** | **QMEAN** | **Sequence Identity (%)** | **Sequence Similarity** | **Coverage** | **Resolution (Å)** |
| --- | --- | --- | --- | --- | --- | --- |
| 6jyt | 0.98 | -5.63 | 99.83 | 0.61 | 1 | 2.8 |
| 5wwp | 0.83 | -1.72 | 72.2 | 0.53 | 0.98 | 3 |
| 6sje | 0.29 | -5.70 | 19.48 | 0.29 | 0.51 | - |
| 4non | 0.28 | -6.79 | 20.32 | 0.29 | 0.52 | 2.00 |
| 5ftf | 0.12 | -5.72 | 17.82 | 0.29 | 0.29 | 2.41 |

**Table S3: Verification of stereochemical quality of SARS-CoV-2 helicase template, modelled and minimised modelled structure**

|  | **PROCHECK (%)** | | | | **G-Factor (°)** | | |
| --- | --- | --- | --- | --- | --- | --- | --- |
|  | **Most favoured** | **Additional allowed** | **Generously allowed** | **Disallowed** | **Torsion angle** | **Covalent geometry** | **Overall average** |
| 5wwp.a | 81.9 | 18.1 | 0 | 0 | -0.41 | 0.48 | -0.06 |
| helicase | 88 | 11.4 | 0.4 | 0.2 | -0.31 | 0.07 | -0.14 |
| min. helicase | 90.9 | 8.3 | 0.6 | 0.2 | 0.04 | -1.38 | -0.63 |

**Table S4: Virtual screening result of molecules against multiple SARS-CoV-2 targets using iGEMDOCK.**

| **S/No.** | **ZINC ID/Potential Drugs** | | **PLpro** | | **3CLpro** | **Helicase** | **RdRp** | **2OMT** | **S-RBD** | **ACE2** | **TMPRSS2** |  |
| --- | --- | --- | --- | --- | --- | --- | --- | --- | --- | --- | --- | --- |
|  | |  | | **Total Energy (kcal/mol)** | | | | | | | | |
|  | ZINC1411 | | -71.4881 | | -65.6971 | -64.7381 | -53.7601 | -73.9287 | -48.9373 | -50.8032 | -75.5047 |  |
|  | ZINC1504 | | -76.1443 | | -82.3289 | -74.7529 | -68.1767 | -77.9059 | -62.3528 | -56.4893 | -89.2018 |  |
|  | ZINC2054 | | -63.9585 | | -62.7038 | -66.9338 | -53.8994 | -64.3134 | -45.7838 | -46.4800 | -68.2458 |  |
|  | ZINC13245 | | -65.9231 | | -67.9089 | -77.0114 | -56.0486 | -72.8733 | -54.8630 | -49.0378 | -78.4909 |  |
|  | ZINC13246 | | -72.4335 | | -72.8918 | -77.7924 | -60.6963 | -75.9899 | -59.9282 | -49.1558 | -80.1536 |  |
|  | ZINC39811 | | -70.5587 | | -66.9718 | -71.5713 | -57.5975 | -68.8462 | -57.3481 | -48.4978 | -72.5629 |  |
|  | ZINC56792 | | -93.1748 | | -93.2962 | -73.2432 | -70.5111 | -100.203 | -67.1040 | -71.8764 | -72.1915 |  |
|  | ZINC57731 | | -84.4144 | | -78.6860 | -68.4397 | -67.3029 | -89.0182 | -57.8100 | -58.5362 | -91.1276 |  |
|  | ZINC57733 | | -85.4932 | | -72.9290 | -80.8632 | -61.5399 | -82.8234 | -55.2274 | -54.7883 | -87.8409 |  |
|  | ZINC119983 | | -91.8904 | | -94.3794 | -79.5733 | -74.4373 | **-101.186** | **-78.1381** | -66.3233 | -97.9281 |  |
|  | ZINC119988 | | -92.6320 | | -91.7674 | -78.7731 | -76.7371 | -88.1754 | -71.1942 | -69.1074 | **-110.077** |  |
|  | ZINC120283 | | -84.0580 | | -83.4596 | -84.9813 | -59.5936 | -80.8573 | -54.8781 | -54.8815 | -89.0955 |  |
|  | ZINC135449 | | **-110.304** | | -98.7905 | -73.6259 | -71.1548 | -93.2562 | **-74.0581** | -65.0318 | -75.5973 |  |
|  | ZINC607790 | | -90.8487 | | -92.3566 | -74.5241 | -77.8366 | **-114.382** | -65.3443 | **-75.4132** | -87.9042 |  |
|  | ZINC607911 | | -94.4299 | | **-105.139** | -76.2577 | -77.0506 | -81.4782 | -68.8746 | -62.8635 | -73.7244 |  |
|  | ZINC1529208 | | -63.5243 | | -57.9630 | -52.2667 | -49.8338 | -57.8240 | -51.2224 | -51.8627 | -63.8938 |  |
|  | ZINC1530009 | | **-127.014** | | **-107.921** | **-92.7142** | **-93.2623** | -99.0911 | -69.5644 | **-85.9533** | **-119.431** |  |
|  | ZINC1530331 | | -54.5082 | | -45.6503 | -50.0660 | -38.3427 | -48.0747 | -41.5303 | -35.5357 | -49.7183 |  |
|  | ZINC1530775 | | -94.1257 | | -75.1813 | **-97.7324** | -70.5176 | **-103.671** | -52.9610 | **-73.5571** | -96.4906 |  |
|  | ZINC1532860 | | -73.1683 | | -70.7127 | -56.8196 | -56.0562 | -62.4018 | -50.6260 | -53.7716 | -64.7894 |  |
|  | ZINC1535009 | | -84.8486 | | -67.5870 | -73.2642 | -69.0346 | -90.1557 | -69.4560 | -57.6340 | -84.9053 |  |
|  | ZINC1597393 | | **-105.863** | | **-109.386** | **-88.0231** | -77.6039 | **-106.249** | -66.8718 | **-80.6556** | **-110.216** |  |
|  | ZINC1618678 | | -94.7463 | | -90.1373 | -83.2197 | -75.1848 | -100.003 | -60.9178 | -71.9460 | -95.1162 |  |
|  | ZINC1697403 | | -51.3229 | | -45.1053 | -49.3447 | -38.9641 | -51.4130 | -36.3285 | -39.4932 | -48.8224 |  |
|  | ZINC1721693 | | -87.8240 | | **-106.044** | -77.1965 | -74.7690 | **-104.113** | -70.9772 | -64.7885 | **-103.386** |  |
|  | ZINC2040970 | | -78.1914 | | -67.6605 | -59.9875 | -58.0946 | -68.6171 | -48.1414 | -49.0840 | -72.0386 |  |
|  | ZINC3824868 | | -93.4912 | | **-101.638** | -86.1088 | -75.3282 | **-101.291** | -67.2569 | -56.3290 | **-120.720** |  |
|  | ZINC3830747 | | -83.2871 | | **-109.463** | -78.1060 | -78.3987 | -88.5525 | -63.1571 | -66.8316 | -93.0868 |  |
|  | ZINC3861087 | | -70.5900 | | -64.2834 | -59.4245 | -62.6556 | -73.0609 | -48.1736 | -53.0517 | -73.2053 |  |
|  | ZINC3869685 | | -99.7823 | | **-114.493** | -86.4292 | **-91.0905** | **-108.898** | -69.3932 | -68.1875 | -82.6934 |  |
|  | ZINC3871576 | | -91.7423 | | **-103.179** | -82.6216 | -76.9380 | -95.6258 | -71.6161 | -65.3703 | -87.1297 |  |
|  | ZINC3978503 | | **-119.764** | | **-122.363** | **-96.3176** | **-104.710** | **-113.965** | **-75.8552** | **-75.1059** | **-117.558** |  |
|  | ZINC5085289 | | **-103.793** | | **-112.414** | **-105.299** | **-99.0848** | **-109.500** | **-78.4112** | **-91.4829** | **-117.445** |  |
|  | ZINC5430812 | | **-104.658** | | **-108.921** | -86.0936 | -79.7586 | -93.6676 | **-77.3572** | **-74.2999** | -86.0940 |  |
|  | ZINC5758645 | | -67.4859 | | -67.8386 | -66.0990 | -60.1458 | -72.5244 | -54.0518 | -53.5651 | -67.6742 |  |
|  | ZINC5783606 | | -94.3203 | | -95.5324 | -74.6393 | -63.8995 | -88.0254 | -61.6801 | -58.8642 | -96.5224 |  |
|  | ZINC5854400 | | -97.0628 | | **-111.538** | **-90.2114** | **-83.6950** | -100.392 | **-75.7087** | **-74.2768** | -98.6792 |  |
|  | ZINC6050191 | | **-97.6645** | | -94.2817 | -80.3503 | **-80.0694** | -84.0333 | -70.1015 | -63.9794 | -94.4306 |  |
|  | ZINC6523722 | | -91.2857 | | -89.6888 | -74.4494 | -69.1148 | -88.9846 | -58.1164 | -61.7330 | -95.9126 |  |
|  | ZINC6524916 | | -88.2256 | | -91.9948 | **-96.1083** | -65.6694 | -94.0899 | -69.5305 | -61.0372 | -99.8796 |  |
|  | ZINC6920379 | | -78.9276 | | -74.4716 | -72.3476 | -59.3566 | -72.5848 | -54.5132 | -58.2369 | -72.7123 |  |
|  | ZINC13282986 | | -97.4831 | | -90.6073 | -73.2262 | -69.6873 | -92.3005 | -60.2770 | -62.1537 | -81.1540 |  |
|  | ZINC13382495 | | **-100.024** | | 156.2900 | **-95.8655** | **-96.3214** | **-101.807** | **-76.8715** | -68.2634 | 268.7460 |  |
|  | ZINC13393275 | | -78.7562 | | -82.8247 | -79.1854 | **-81.9464** | -79.9239 | -59.1748 | -68.1149 | -100.865 |  |
|  | ZINC13512214 | | -61.5690 | | -61.2930 | -60.7688 | -50.2946 | -60.9247 | -40.8449 | -43.2093 | -64.2684 |  |
|  | ZINC13539005 | | **-101.037** | | -93.6820 | **-90.5302** | **-86.1649** | -100.511 | -70.2349 | -69.1194 | **-107.341** |  |
|  | ZINC14586037 | | -88.0137 | | -96.1151 | -73.6857 | **-86.7963** | -97.3296 | -57.5840 | -61.5797 | -74.2830 |  |
|  | ZINC14642643 | | **-123.048** | | -98.8200 | **-95.5790** | **-91.9700** | -86.1965 | **-71.7790** | **-75.3058** | **-110.325** |  |
|  | ZINC14689062 | | **-105.305** | | -98.3196 | -84.2110 | -76.3324 | -97.0051 | **-74.2555** | **-76.7180** | **-107.985** |  |
|  | ZINC14768737 | | **-102.915** | | -93.3995 | -82.1971 | **-102.771** | **-103.034** | **-72.6260** | **-74.6495** | -89.7831 |  |
|  | ZINC14819573 | | -92.6219 | | -97.6188 | **-89.1064** | -73.8379 | -89.4035 | **-77.1362** | -66.6123 | -92.9019 |  |
|  | ZINC15043216 | | **-105.155** | | **-100.389** | -89.3112 | **-107.434** | **-106.213** | **-81.2413** | **-89.9787** | **-133.244** |  |
|  | ZINC15118974 | | **-97.6526** | | -86.4115 | **-96.1425** | -76.5556 | -90.2838 | -68.8883 | -68.4397 | **-107.289** |  |
|  | ZINC18157343 | | -63.6434 | | -63.1362 | -63.6305 | -45.9394 | -63.9877 | -45.7662 | -41.6261 | -72.9572 |  |
|  | ZINC18185774 | | -96.7987 | | **-101.626** | -84.5143 | **-89.7451** | **-104.123** | **-77.9968** | **-73.2407** | -93.9804 |  |
|  | ZINC18825330 | | -85.1768 | | -97.9094 | -82.7938 | -71.8046 | **-105.008** | -62.0838 | -67.2907 | **-107.005** |  |
|  | ZINC18847044 | | -90.8265 | | -80.6574 | **-91.8354** | -73.7491 | -97.4931 | -65.1841 | -66.7897 | **-115.542** |  |
|  | ZINC18847046 | | -90.1628 | | -74.6832 | -87.4986 | -67.2511 | -85.3441 | -59.7236 | -57.2808 | -94.6135 |  |
|  | ZINC28109109 | | -84.1920 | | -76.7942 | -71.2139 | -68.6161 | -78.8965 | -59.0766 | -57.9433 | -93.4922 |  |
|  | ZINC30726889 | | -79.8151 | | -76.3775 | -75.0411 | -58.3228 | -70.0127 | -52.2231 | -60.3375 | -75.7764 |  |
|  | ZINC33831297 | | -60.2871 | | -77.1916 | -76.4625 | -64.7585 | -74.6642 | -62.7583 | -59.8272 | -77.3909 |  |
|  | ZINC38143792 | | -78.1386 | | -88.6675 | 71.18020 | -79.9719 | -29.8219 | **-71.7659** | -54.2188 | -79.9557 |  |
|  | ZINC40422816 | | **-109.714** | | **-107.341** | **-96.7958** | **-91.0584** | **-114.424** | **-72.7935** | **-75.6718** | **-108.829** |  |
|  | ZINC53194131 | | -92.0599 | | -87.3372 | -70.7983 | -67.0592 | -80.4933 | -62.6551 | -59.1542 | -89.1420 |  |
|  | ZINC95619153 | | -92.4591 | | -97.9804 | **-89.1751** | -78.8235 | -84.5214 | -62.5859 | **-87.6877** | -93.8631 |  |
|  | Chloroquine | | -77.0561 | | -85.2894 | -70.2726 | -61.2529 | -76.8853 | -61.0419 | **-56.6653** | -89.6391 |  |
|  | Hydroxychloroquine | | -89.5946 | | -90.9167 | -70.1326 | -63.0921 | -86.1140 | -57.0905 | **-65.1659** | **-95.6860** |  |
|  | Lopinavir | | **-124.236** | | -82.8801 | -79.0957 | -96.4897 | -85.3790 | **-68.5646** | -47.7620 | - |  |
|  | Remdesivir | | **-99.3610** | | **-96.8100** | **-94.5728** | **-103.548** | **-103.269** | **-67.1600** | -56.4427 | - |  |
|  | Ritonavir | | -91.5763 | | **-106.972** | **-90.7169** | **-97.3424** | **-101.456** | -50.9849 | -45.5257 | - |  |
|  | Camostat mesylate | | - | | - | - | - | - | - | - | -91.7173 |  |
|  | Nafamostat mesylate | | - | | - | - | - | - | - | - | **-92.7771** |  |
|  | bromhexine hydrochloride | | - | | - | - | - | - | - | - | -72.2068 |  |

PLpro, 3CLpro, RdRp, 2OMT, S-RBD, ACE2 and TMPRSS2 represent papain like protease, main/3-chymotrypsin-like protease, RNA-dependent RNA polymerase, 2-O-methyltransferase, spike receptor binding domain, human angiotensin-converting enzyme 2 and human type-II transmembrane serine protease respectively. Top 15 molecules and top 2 standards respectively are emboldened.
